# Metabolic Flux Analysis of Catechin Biosynthesis Pathways Using Nanosensor

**DOI:** 10.3390/antiox9040288

**Published:** 2020-03-31

**Authors:** Habiba Kausar, Ghazala Ambrin, Mohammad K. Okla, Walid Soufan, Abdullah A. Al-Ghamdi, Altaf Ahmad

**Affiliations:** 1 Department of Botany, Aligarh Muslim University, Aligarh 202002, India; hkausar@myamu.ac.in (H.K.); ambringhazala@gmail.com (G.A.); 2 Botany and Microbiology Department, College of Science, King Saud University, P.O. Box. 2460, Riyadh 11451, Saudi Arabia; malokla@ksu.edu.sa (M.K.O.); abdaalghamdi@ksu.edu.sa (A.A.A.-G.); 3 Plant Production Department, Faculty of Food and Agricultural Sciences, King Saud University, P.O. Box 2460, Riyadh 11451, Saudi Arabia; wsoufan@ksu.edu.sa

**Keywords:** (+)-catechin, fluxomics, nanosensor, green tea

## Abstract

(+)-Catechin is an important antioxidant of green tea (*Camelia sinensis* (L.) O. Kuntze). Catechin is known for its positive role in anticancerous activity, extracellular matrix degradation, cell death regulation, diabetes, and other related disorders. As a result of enormous interest in and great demand for catechin, its biosynthesis using metabolic engineering has become the subject of concentrated research with the aim of enhancing (+)-catechin production. Metabolic flux is an essential concept in the practice of metabolic engineering as it helps in the identification of the regulatory element of a biosynthetic pathway. In the present study, an attempt was made to analyze the metabolic flux of the (+)-catechin biosynthesis pathway in order to decipher the regulatory element of this pathway. Firstly, a genetically encoded fluorescence resonance energy transfer (FRET)-based nanosensor (FLIP-Cat, fluorescence indicator protein for (+)-catechin) was developed for real-time monitoring of (+)-catechin flux. *In vitro* characterization of the purified protein of the nanosensor showed that the nanosensor was pH stable and (+)-catechin specific. Its calculated *K*_d_ was 139 µM. The nanosensor also performed real-time monitoring of (+)-catechin in bacterial cells. In the second step of this study, an entire (+)-catechin biosynthesis pathway was constructed and expressed in *E. coli* in two sets of plasmid constructs: pET26b-PT7-rbs-PAL-PT7-rbs-4CL-PT7-rbs-CHS-PT7-rbs-CHI and pET26b-T7-rbs-F3H-PT7-rbs- DFR-PT7-rbs-LCR. The *E. coli* harboring the FLIP-Cat was transformed with these plasmid constructs. The metabolic flux analysis of (+)-catechin was carried out using the FLIP-Cat. The FLIP-Cat successfully monitored the flux of catechin after adding tyrosine, 4-coumaric acid, 4-coumaroyl CoA, naringenin chalcone, naringenin, dihydroquercetin, and leucocyanidin, individually, with the bacterial cells expressing the nanosensor as well as the genes of the (+)-catechin biosynthesis pathway. Dihydroflavonol reductase (DFR) was identified as the main regulatory element of the (+)-catechin biosynthesis pathway. Information about this regulatory element of the (+)-catechin biosynthesis pathway can be used for manipulating the (+)-catechin biosynthesis pathway using a metabolic engineering approach to enhance production of (+)-catechin.

## 1. Introduction

Antioxidants are compounds that are intrinsic to the defence system of living cells. They are synthesized by cells against the negative impact of free radicals. The synthesis of antioxidants is controlled by a complex framework inclusive of both enzymatic and non-enzymatic pathways and is inherent to both animals and plants. Apart from the inmost capability of antioxidant synthesis, cells can also take up antioxidants through exogenous supply, either in the form of eating habits or through dietary supplements [1,2,3,4]. (+)-Catechin is one of the most popular and powerful sources of antioxidants. It is also recognized for antihypertensive, anti-diabetic, anti-thrombogenic, and anti-proliferative activities and has a vital role to play in the molecular mechanisms of cell death regulation, extracellular matrix degradation, and angiogenesis [5,6,7,8,9,10]. Synergistic anticancer activity of catechin along with caffeine on different cancer cells has been demonstrated [11]. In plants, (+) catechin acts as an infection inhibiting factor and an allelochemical [12]. Owing to the properties of (+)-catechin, especially the most consumer fascinating antioxidant property, a remarkable upsurge in its demand has been recorded. The main biological source of the (+)-catechin is green tea [*Camelia sinensis* (L.) O. Kuntze]. Therefore, green tea is exploited to produce the marketed forms of (+)-catechin, i.e., tablets and dietary supplements [13]. Moreover, reports state that the absorption rate of catechin is very low, which means that the intake of (+)-catechin should be in a more substantial amount to reach an efficient concentration [14].

**Figure 1:**
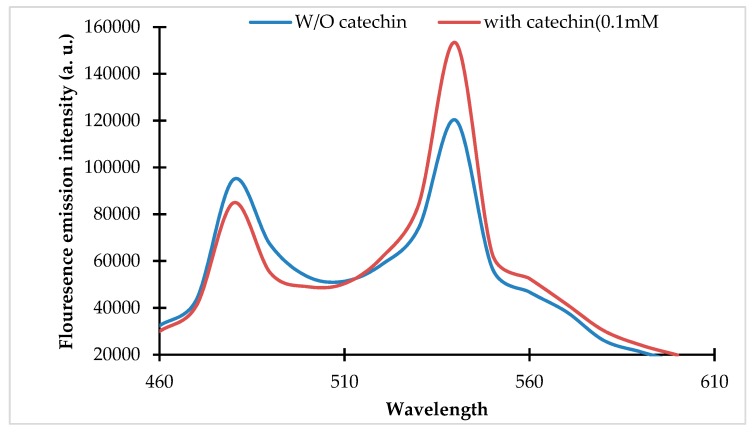
Spectral analysis of the purified protein of the fluorescence indicator protein for (+)-catechin (FLIP-Cat) nanosensor. Fluorescence emission intensity (460 nm to 610 nm) was recorded by the addition of (+)-catechin (0.1 mM) and without (+)-catechin (0 mM). Excitation of FLIP-Cat was carried out at 430 nm.

Because of the large demand of (+)-catechin, its biosynthesis has become the subject of intensive research so that production of (+)-catechin can be enhanced through a metabolic engineering approach. Enzyme-encoding genes of (+)-catechin biosynthesis pathways have been identified, and a pathway has been proposed that starts either from tyrosine to form 4-coumaric acid or from phenylalanine forming cinnamic acid. The reactions are catalyzed using tyrosine ammonia-lyase (TAL) and phenylalanine ammonia-lyase (PAL), respectively. The (+)-catechin is then synthesized through six enzymatic steps, as shown in Figure 1, involving chalcone synthase (CHS), chalcone isomerase (CHI), flavanone 3b-hydroxylase (F3H), dihydroflavonol reductase (DFR) and leuacoanthocyanidin reductase (LCR) [15,16]. However, the studies on regulation of catechin biosynthesis are inconclusive. Knowledge of the regulation of the metabolic pathway prior to using a metabolic engineering approach has become more important. This avoids the need to generate large numbers of transgenic plants on a trial-and-error basis to test different intervention strategies [17]. As the metabolic network associated with the biosynthesis of the compounds is highly complex and interconnected, the study of such intricate pathways requires fast and systematic methodologies [18]. Fluxomics analysis of a metabolic pathway promises to identify the regulatory element affecting the flux of metabolites in a metabolic pathway. The flux of metabolites through a metabolic network depends on the rate-limiting step. This rate-limiting step can, therefore, be the key target for metabolic engineering. One of the best approaches for the identification of the key regulatory step of a metabolic network is to screen the production of metabolites after metabolic engineering. Genetically encoded nanosensors that transduce intracellular metabolite concentration into gene expression changes enable the best screening of metabolite production through metabolic engineering in real time by coupling the production of metabolites with the expression of fluorescent proteins. These nanosensors possess the capability to study the real-time dynamics of the target analyte even in single cells. The tool is non-invasive and has excellent spatial and temporal resolution [19,20]. At present, there are many fluorescence resonance energy transfer (FRET)-based nanosensors developed for specific analytes like glutamine, histidine, vitamin B12, lysine, arginine, and maltose [20,21,22,23,24,25].

In the present study, firstly, a genetically encoded nanosensor (fluorescence indicator protein for (+)-catechin, FLIP-Cat) was developed for the real-time monitoring of (+)-catechin in living cells. Thereafter, the entire phenylpropanoid pathway (phenylalanine ammonia-lyase, PAL; 4-coumarate: CoA ligase, 4CL; chalcone synthase, CHS; chalcone isomerase, CHI; flavanone 3 hydroxylase, F3H; dihydroflavonol reductase, DFR; leuacoanthocyanidin reductase, LCR) was constructed (Supplementary Figure S1) and expressed in *Escherichia coli* as the function of the genes, which can be studied in bacteria that lack certain endogenous enzymes [26]. Thereafter, fluxes on metabolites of the (+)-catechin biosynthesis pathway were analyzed using a FRET-based genetically encoded nanosensor. This study promises to study the single cell dynamics of (+)-catechin production and identify the regulatory gene affecting its synthesis and, thereby, provides an opportunity to improve the production and yield of (+)-catechin.

## 2. Materials and Methods

### 2.1. Development of Nanosensor

#### 2.1.1. Designing and Construction

A (+)-catechin binding protein, fraa-3 from *Fragaria ananassa*, was used as a ligand sensing element for the construction of a catechin sensor. The protein structure of fraa-3 was studied on the Protein data bank (PDB) database. The complete nucleotide sequence of fraa-3 was retrieved from The National Center for Biotechnology (NCBI) (Accession No-GQ148819). The fraa-3 cDNA was obtained from Prof. Victoriano Valpuesta, Biologia Molecular Y Bioquimica, Universidad De Malaga, Spain as a gift [27]. Once the sensing element was chosen, variants of green fluorescent proteins were selected to construct the sensor. Enhanced cyan fluorescent protein (ECFP) as donor and Venus (a yellow fluorescent protein) as acceptor fluorophore were used as the FRET pair. The sensor was designed to attach the donor fluorophore at the N-terminus of the fraa-3 protein and the acceptor fluorophore at the C-terminus of the fraa-3 protein. The genes encoding the ECFP, Venus and fraa-3 protein were amplified using PCR with using forward and reverse primers. The sequences of the primers for amplification of ECFP, Venus and fraa-3 are given in Supplementary Table S1. Bold sequences show the restriction sites. Stop codons were avoided during the designing of primers. The ECFP and Venus were cloned in pGEMT^®^-T easy vector (Promega, Madison, WI, USA). Thereafter, the fraa-3 gene was inserted in between the ECFP/Venus cassette to yield the ECFP-Fraa3-Venus construct, cloned in pGEM^®^-T easy vector (Promega, Madison, WI, USA), generating pGEMT_ECFP-Fraa3-Venus. This construct was then sub-cloned into the pRSET-B bacterial expression vector (Novagen, Dermstadt, Germany), generating pRSET_ECFP-Fraa3-Venus. Schematic representations of the pGEMT_ECFP-Fraa3-Venus and pRSET_ECFP-Fraa3-Venus are shown as Supplementary Figure S2 and Figure S3, respectively. The pRSET-B vector adds an in-frame (His)_6_ tag at the amino terminus of the self-synthesized protein, which allows the recombinant protein to be isolated through Ni-NTA Histidine-tag affinity chromatography. The developed construct of the sensor was named FLIP_Cat (fluorescent indicator protein for (+)-catechin). Further, by employing the electroporation technique, the construct (pRSET-ECFP_Fraa 3_Venus) was introduced into *E. coli* BL21 (DE3). To affirm the sensor fidelity, chimeric fragment was sequenced.

#### 2.1.2. Expression and Purification of Sensor Protein

The *E. coli* BL21 (DE3) cells transformed with pRSET-ECFP_Fraa3_Venus were grown in Luria Bertani medium for 24 h at 20 °C upto O.D._600_ 0.6, followed by the induction of 0.5 mM isopropyl β-D-1 thiogalactopyranasoide for the expression of the sensor protein. After induction, cells were allowed to grow in the dark for 48 h at 20 °C for the production of sensor protein. Cells were then harvested by centrifugation for 20 min at 6500× *g*. The supernatant was discarded, and the cell pellet was resuspended in 20 mM Tris-Cl, pH 8.0. The bacterial cells were disrupted using ultrasonication to isolate the proteins. For proper folding, eluted proteins were stored overnight at 4 °C.

#### 2.1.3. Characterization of Sensor Protein

The purified sensor protein was characterized to know the working capacity of the sensor. The characterization of the sensor was carried out in terms of FRET occurrence, pH stability, specificity and affinity. Initially, spectral analysis of the purified protein of a FLIP-Cat nanosensor was carried out by exciting the ECFP at 430 nm, followed by measurement of emission intensities in the range of 450–610 nm after adding 0.1 mM (+)-catechin. A spectrum was also taken without (+)-catechin under similar conditions. To investigate the pH stability of the sensor protein, the FRET ratio (Venus/ECFP emission intensity ratio) was measured using different buffer systems with different pH values after adding 0.1 mM of (+)-catechin. Phosphate buffer saline (PBS), Tris-HCl, Tris-buffered saline (TBS), and 3-(N-morpholino) propanesulphonic acid (MOPS), each of pH range 5–7, were taken. A microplate reader (Synergy H1, Biotek, Winooski, VE, USA) was employed to monitor the change in the Venus/ECFP emission intensity ratio with respect to changes in buffer and pH.

Sensor specificity analysis was performed with different antioxidant metabolites, (+)-catechin, (−)-epicatechin, (−)-epigallocatechin, (−)-epigallocatechin gallate, anthocyanin, quercetin, dihydroquercetin, and leucocyanidin at 0, 1.0 mM, and 10 mM each. To carry out the measurements, 180 µL sensor protein and 20 µL substrate were taken in each well of a microplate. Emission intensities of ECFP and Venus were measured at 485/20 nm and 535/25 nm for ECFP and Venus, respectively, after excitation at 430/20 nm using a microplate reader. For calculating the affinity of the sensor, the ratio of the emission intensities of ECFP and Venus, measured at different concentrations of catechin and binding constant (*K*_d_) of the sensor, was determined by fitting the ligand ((+)-catechin) titration curve into the simple binding isotherm:

S = (r−r_apo_)/(r_sat_−r_apo_) = [L]/(*K*_d_+[L]), where S is saturation; [L] is ligand concentration; r is ratio; r_apo_ is ratio in the absence of ligand; r_sat_ is ratio at saturation with the ligand. All experiments were performed with at least three independent protein samples.

The pRSET_FLIP-Cat transformed *E. coli* BL21 cells were grown in LB medium at 20 °C for 72 h and then stored at 4 °C overnight to allow the proper folding of the sensor protein. Cells were harvested using centrifugation and adjusted to OD_600_ = 1.5, followed by dissolving the harvested cell pellet in 20 mM MOPS buffer (pH 7.5). 180 µL of cells were added in each well of a microplate, followed by the addition of 20 µL 0.1 mM (+)-catechin. Emission intensities were measured at 485 nm/20 nm and 535 nm/25 nm for ECFP and Venus, respectively after excitation at 430 nm/20 nm. The FRET ratio (ratio of emission intensities of Venus/ECFP) was calculated. Measurement of the FRET ratio was recorded at regular intervals for 30 min. Specificity of the FLIP-Cat was also measured in pRSET_FLIP-Cat-expressed *E. coli* BL21 cells using (−)-epicatechin, (−)-epigallocatechin, (−)-epigallocatechin gallate, anthocyanin quercetin, dihydroquercetin, and leucocyanidin.

### 2.2. Construction and Expression of (+)-Catechin Biosynthetic Pathway

#### 2.2.1. Construction

The complete (+)-catechin biosynthesis pathway was divided into two sets of plasmid, pET26b-PT7-rbs-PAL-PT7-rbs-4CL-PT7-rbs-CHS-PT7-rbs-CHI and pET26b-T7-rbs-F3H-PT7-rbs- DFR-PT7-rbs-LCR, following the earlier established methods of Umar et al. [28] and Hwang et al. [16]. For the construction of pET26b-T7-rbs-PAL-T7-rbs-4CL-T7-rb-CHS-T7-rbs-CHI, the nucleotide sequence of the genes were retrieved from the NCBI, except for the 4CL, which was obtained from www.sanger.ac.uk/project/S_coelicolor [29]. PAL and 4CL were isolated from the *Rhodotorula rubra* and *Streptomyces coelicolor*A3(2), while the rest of the genes (CHS, CHI, F3H, DFR, LCR) were isolated from the green tea (*Camelia sinensis*). All the genes were amplified using gene-specific primers with the appropriate restriction sites. The sequences of forward and reverse primers are given in supplementary Table S2. The in-house designed primers of PAL contained sequences of *Nde*I-PT7-rbs/*Msc*I. Similarly, sequences of*Msc*I-PT7-rbs/*Bam*HI, *Bam*HI-PT7-rbs/*Sac*I, and *Sac*I-PT7-rbs/*Hin*dIII were added with the primers of 4CL, CHS, and CHI, respectively. To facilitate the restriction cloning in the pET26b vector (Novagen, Darmstadt, Germany), all the genes were initially cloned in the pGEMT-easy vector, resulting in pGEMT_*Nde*I-PT7-rbs/*Msc*I_PAL, pGEMT_*Msc*I-T7-rbs/*Bam*HI_4CL, pGEMT_*Bam*HI-T7-rbs/*Sac*I_CHS, and pGEMT_*Sac*I-T7-rbs/*Hin*dIII_CHI. The *Nde*I-PT7-rbs/*Msc*I_PAL fragment of pGEMT-*Nde*I-PT7_rbs/*Msc*I_PAL was cloned into pET26b at *Nde*I/*Msc*I sites resulting in pET26b-T7-rbs-PAL. In the same manner, *Msc*I-T7-rbs/*Bam*HI_4CL, *Bam*HI-PT7-rbs/*Sac*I_CHS, *Sac*I-PT7-rbs/*Hin*dIII_CHI fragments of pGEMT_*Msc*I-T7-rbs/*Bam*HI_4CL, pGEMT_*Bam*HI-PT7-rbs/*Sac*I_CHS, and pGEMT_*Sac*I-PT7-rbs/*Hin*dIII_CHI, respectively, were cloned into pET26b-PT7-rbs-PAL stepwise at corresponding sites to form pET26b-PT7-rbs-PAL-PT7-rbs-4CL-PT7-rb-CHS-PT7-rbs-CHI (Supplementary Figure S4). The same approach was followed for constructing the pET26b-PT7-rbs-F3H-PT7-rbs-DFR-PT7-rbs-LCR plasmid firstly. These were cloned in the pGEMT-easy vector, followed by restriction cloning in the pET26b vector. The in-house designed primers (Supplementary Table S2) of F3H, DFR, and LCR contained sequences of *Nde*I-PT7-rbs/*Sac*I, *Sac*I-PT7-rbs/*Hin*dIII, *Hin*dIII-PT7-rbs/*Not*I, respectively. Following the same strategies, before cloning into the pET26b vector, all the genes were cloned in the pGEMT-easy vector resulting in pGEMT_*Nde*I-PT7-rbs/*SacI*_F3H, pGEMT_*Sac*I-PT7-rbs/*Hin*dIII_DFR, and pGEMT_*Hin*dIII-PT7-rbs/*Not*I_LCR constructs. These genes were then introduced into the pET26b vector sequentially by gene and site-specific restriction digestion. The fragment *Nde*I-PT7-rbs/*Sac*I_F3H derived from pGEMT_*Nde*I-PT7-rbs/*Sac*I_F3H was cloned into pET26b at the *Nde*I/*Sac*I site by restriction digestion formingpET26b-PT7-rbs-F3H construct, followed by cloning of *Sac*I-PT7-rbs/*Hin*dIII_DFR and *Hin*dIII-PT7-rbs/*Not*I_LCR fragments of pGEMT_*Sac*I-PT7-rbs/*Hin*dIII_DFR and pGEMT_*Hin*dIII-PT7-rbs/*Not*I_LCR into pET26b-PT7-rbs-F3H at *Sac*I/*Hin*dIII and *Hin*dIII/*Not*I, respectively, resulting in pET26b-PT7-rbs-PAL-PT7-rbs-4CL-PT7-rb-CHS-PT7-rbs-CHI construct (Supplementary Figure S5).

#### 2.2.2. Expression

The *E. coli* cells were transformed separately with pET26b-PT7-rbs-PAL-PT7-rbs-4CL-PT7-rbs- CHS-PT7-rbs-CHI and pET26b-PT7-rbs-F3H-PT7-rbs-DFR-PT7-rbs-LCR and also with a combination of these two plasmids. Based on the transformation, the primary culture of the bacterial cells harboring plasmids was prepared. The *E. coli* cells having pET26b-PT7-rbs-PAL-PT7-rbs-4CL-PT7-rbs-CHS-PT7-rbs-CHI, pET26b-PT7-rbs-F3H-PT7-rbs-DFR- PT7-rbs-LCR and both the plasmids were allowed to grow overnight in 10 mL of LB media with continuous shaking at 37 °C. The next morning, three flasks having freshly prepared 100 mL of LB liquid media were taken, and from each primary culture, 2 mL was added to the fresh LB media, respectively. The cultures were incubated in a shaker at 37 °C and were allowed to grow until the O.D._600_ of the cultures reached 0.6. At this stage, flasks were capped and sealed. To induce protein expression, 1 mM IPTG was added to the culture.

#### 2.2.3. Validation

After inducing the protein expression, culture expressing pET26b-T7-rbs-PAL-4CL-CHS-CI and combination plasmids were supplemented with 0.1 mM tyrosine, while the culture harboring pET26b-T7-rbs-F3H-DFR-LAR was supplemented with 0.2 mM naringenin, respectively. The cultures were allowed to grow in an incubator shaker for 72 h at 30 °C, and High performance liquid chromatography (HPLC) analysis was performed to validate the naringenin and (+)-catechin produced by the expressed plasmids. For HPLC analysis, the extraction method proposed by Hwang et al. [16] and Chemler et al. [30] was adopted. For the extraction of naringenin and (+)-catechin, the broth culture harboring the expressed plasmid for naringenin and (+)-catechin production were taken and adjusted to pH 9.0 with the help of 0.5 mM NaOH. After which the cultures were allowed to stand at room temperature for 1 h. This was followed by the extraction of the produced compounds in the culture with equal volumes of ethyl acetate, which resulted in the formation of an organic and residual layer. The organic layer was evaporated and the residual layer was dissolved in acetonitrile for HPLC analysis using reverse-phase ZORBAX-SB C18 Column (4.6 mm × 150 mm). The flow rate of solvent gradient was maintained at 1 mL/min. This gradient was created by using solvent A (acetonitrile) and solvent B (water), both containing 0.1% formic acid. The conditions according to which HPLC was programmed were 10–40% A (0–10 min), 40–60% A (10–15 min). Absorbance was monitored at 280 nm. The retention time of the (+)-catechin and naringenin was compared with the standard samples to identify the peaks.

### 2.3. Flux Analysis of Metabolites of Catechin Biosynthesis Pathway

#### 2.3.1. Microplate Analysis

The FLIP_Cat was expressed in *E. coli* BL21 (DE3) cells along with pET26b_PT7-rbs-PAL-4CL-CHS-CHI and pET26b_PT7-rbs-F3H-DFR-LAR as described above. These cells were transferred to a 96-well microplate. Tyrosine (for PAL), 4-coumaric acid (for 4CL), 4-coumaroyl CoA (for CHS), naringenin chalcone (for CHI), naringenin (for F3H), dihydroquercetin (for DFR), and leucocyanidin (for LAR) were added with the bacterial cells in different wells at a concentration of 1 mM. The ratio of emission intensities of Venus and ECFP were recorded for 35 min at regular intervals of 5 min using a microplate reader after excitation. The filer sets were 485 nm/20 nm and 535 nm/25 nm for ECFP and Venus emission intensities, respectively, and 430 nm/20 nm for excitation. Only buffer was added in three wells, which served as the control.

#### 2.3.2. Confocal Analysis

The *E. coli* cells after transformation with the FLIP-Cat and plasmid harboring (+)-catechin biosynthesis gene (pET26b-PT7-rbs-PAL-PT7-rbs-4CL-PT7-rbs-CHS-PT7-rbs-CHI and pET26b-T7-rbs-F3H-PT7-rbs-DFR-PT7-rbs-LCR) were utilized to carry out confocal imaging on a confocal microscope (DMRE, Leica, Wetzlar, Germany). This microscope is equipped with a TCS-SPE confocal head that uses a 63 × water immersion objective lens. LAS-AF software was used to record the dual emission intensity ratio corresponding to catechin flux. Expressed bacterial cells were perfused with different substrates at a flow rate of 1.0 mL/min in a vacuum chamber. Addition of each substrate was followed by washing of the bacterial cell for 2 min to remove the traces of previous substrates.

## 3. Results

### 3.1. Design and Construction of Sensor

The chosen fraa-3 protein is a member of the pathogenesis-related 10 (PR10) protein, which further belongs to the START superfamily. These proteins have the ability to adopt the helix grip fold with an internal cavity, and structural analyses of these proteins have suggested that binding of (+)-catechin induces conformational changes in critical regions, which makes it fit to be used as the reporter element for our sensor. (+)-Catechin binding is associated with the closed conformation of loop L5 and involves both polar and hydrophobic interaction. Previous studies regarding fraa-3-(+)-catechin complex have provided us with the details of the fraa-3 structure, wherein loop L5 folds over one end of the catechin molecule and proceeds towards the C-terminal α-helix (α3) on the contrary side, limiting the access to the cavity. Simultaneously, helix α3 of fraa-3 shows a slight twist at its mid toward loop L5, while loop L3 proceeds towards loop L5 and the ligand. This leads to closed conformation, which is stabilized by interactions with the (+)-catechin molecule [27]. The property of the fraa-3 protein in that it undergoes conformational changes on binding with the (+)-catechin ligand makes it an excellent candidate to develop a nanosensor for measuring the (+)-catechin. In our study, we attached the donor fluorophore (ECFP) at the N-terminus and acceptor fluorophore (Venus) at the C-terminus of the reporter fraa-3 element. Figure S6a portrays the arrangements of the restriction sites in the construct. Figure S6b delineates the functional architecture of the FRET-based sensor. Since the prerequisite of the FRET is the distance between the fluorophores, the fraa-3 protein undergoes conformational changes in the presence of the (+)-catechin, making the fluorescent protein come close and the FRET to occur. The development of the recombinant construct in the pGEMT and pRSET vectors was confirmed using nucleotide sequencing (Supplementary Figure S7) and restriction digestion analysis using the appropriate restriction enzymes (Supplementary Figure S8–S12).

### 3.2. Characterization of the Sensor

The pRSET_FLIP-Cat was transformed and expressed in *E. coli* BL21 cells. The recombinant protein was purified using Ni-NTA affinity chromatography. The purified protein was subjected to emission spectral analysis, which revealed a change in the emission spectra of the ECFP and Venus in the presence of the (+)-catechin (Figure 1).

**Figure 2:**
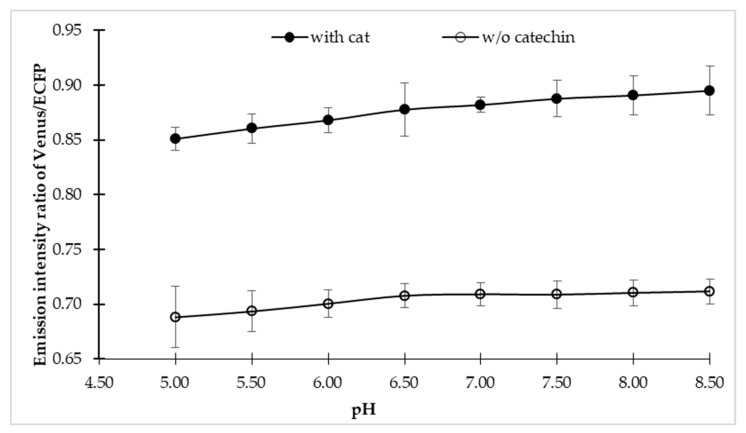
pH stability of FLIP-cat sensor. pH stability of FLIP-Cat was performed by dissolving the purified protein of FLIP-Cat in 3-(N-morpholino) propane sulphonic acid (MOPS) buffer of various pH range (5–8.5) and monitoring the emission intensity ratio of Venus/enhanced cyan fluorescent protein (ECFP). Plotted values are means of three independent replicates. Vertical bar indicates the standard error.

To explore the sensitivity of the sensor in the physiological condition of a cell, fluorescence emission intensities of the sensor were examined in different buffer, each with a pH range of 4.5–8.5, both in the presence and absence of the catechin. The FLIP-Cat sensor exhibited maximum stability in MOPS buffer over the wider pH range. In addition, no notable significant effect of pH change was observed on the sensor stability (Figure 2). Therefore, the MOPS buffer at pH 7.5 was considered to be suitable for further experimental studies. Sensor stability over a wider range of pH is very crucial in any in vivo analysis as the subcellular compartments of the cell may have different pH values.

**Figure 3:**
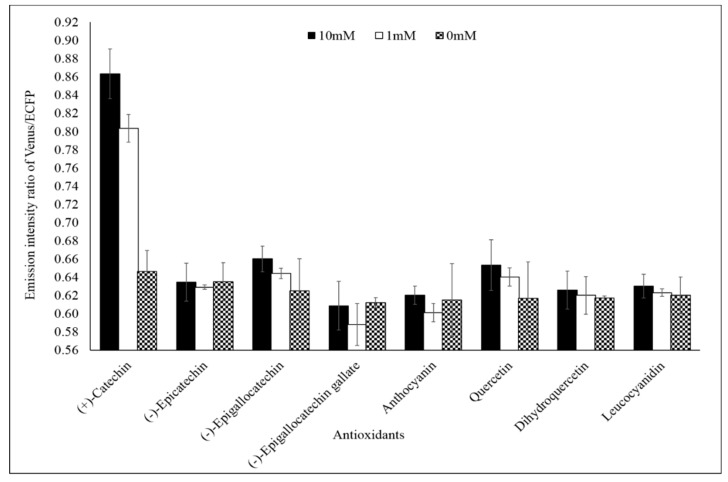
Specificity analysis of FLIP-Cat. Purified nanosensor protein (20 mg/mL) was taken in different wells of a microplate and various homologous metabolites of catechin (0, 1, and 10 mM) were added. In one well (+)-catechin (0, 1, and 10 mM) was added. The fluorescence resonance energy transfer (FRET) ratio was recorded. Values are the means of three independent replicate. Vertical bars show the standard error.

**Figure 4:**
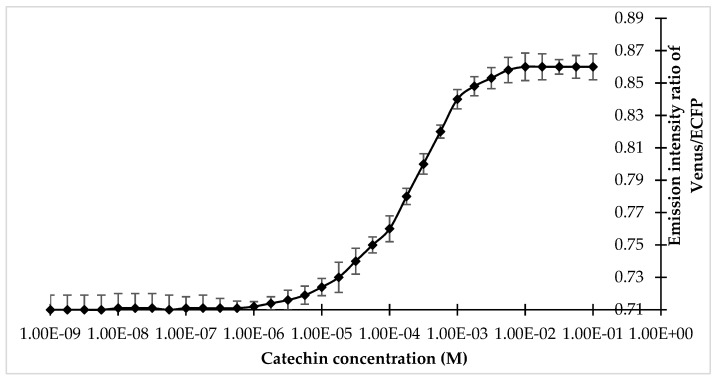
Titration curve of FLIP-Cat at different concentrations of (+)-catechin.Values are means of three independent replicates. Vertical bar indicates standard error.

Specificity analysis of the FLIP-Cat towards (+)-catechin and homologous metabolite (−)-epicatechin, (−)-epigallocatechin, (−)-epigallocatechin gallate, anthocyanin, quercetin, dihydroquercetin, and leucocyanidin at 1 mM and 10 mM showed a significant change in the FRET ratio only in the presence of (+)-catechin. No significant change was observed in the FRET ratio in the case of the other metabolites (Figure 3). All the metabolites were purchased from Sigma-Aldrich, USA. Thus, FLIP-Cat can be viewed as a specific (+)-catechin-sensor appropriate for in vivo imaging. Titration curve analysis of the purified sensor protein at different concentrations of the (+)-catechin (1–100 mM) reported a sigmoidal curve in response to increasing (+)-catechin concentration, which saturated at 10 mM. The calculated affinity of the FLIP-Cat sensor was 139 µM (Figure 4).

**Figure 5:**
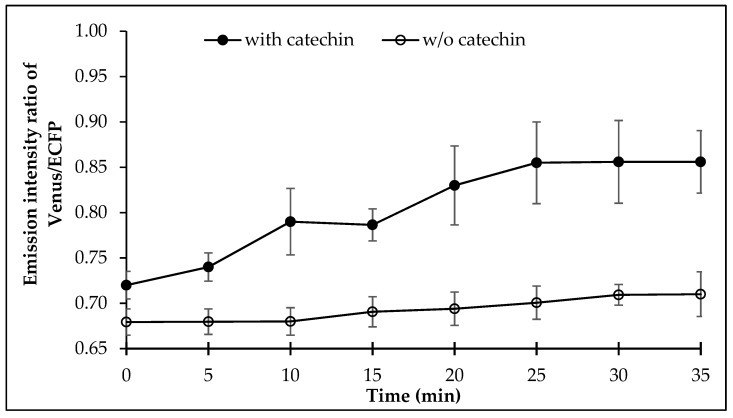
Dynamics of (+)-catechin levels in live bacterial cell. Values are means of three independent replicates. Vertical bar indicates standard error.

Measurement of the FRET in the *E. coli* BL21(DE3) cells expressing FLIP-Cat was examined for FRET after addition of (+)-catechin externally. It was observed that (+)-catechin addition led to a significant change in the FRET ratio of the cell culture, which increased sharply after 7 min, following the (+)-catechin addition externally and saturated after 25 min (Figure 5).

### 3.3. Validation of the (+)-Catechin Biosynthesis Pathway

The HPLC analysis validated the compounds produced by the *E. coli* cells harbouring pET26b-PT7-rbs-PAL-PT7-rbs-4CL-PT7-rbs-CHS-PT7-rbs-CHI and pET26b-PT7-rbs-F3H-PT7-rbs-DFR-PT7-rbs-LCR. The retention time for the standard compound naringenin and (+)-catechin under these conditions were 4 and 16.2, respectively (Supplementary Figure S13 and S14). The retention times of the isolated (+)-catechin and naringenin produced in culture were same as the standard compounds ((+)-catechin and naringenin). The peak obtained in HPLC suggested that no other compounds were produced in culture except naringenin and (+)-catechin. These results suggest successful production of (+)-catechin and naringenin using the developed construct.

### 3.4. Fluxomic Analysis of Catechin Biosynthesis Pathway

#### 3.4.1. Microplate Analysis

**Figure 6:**
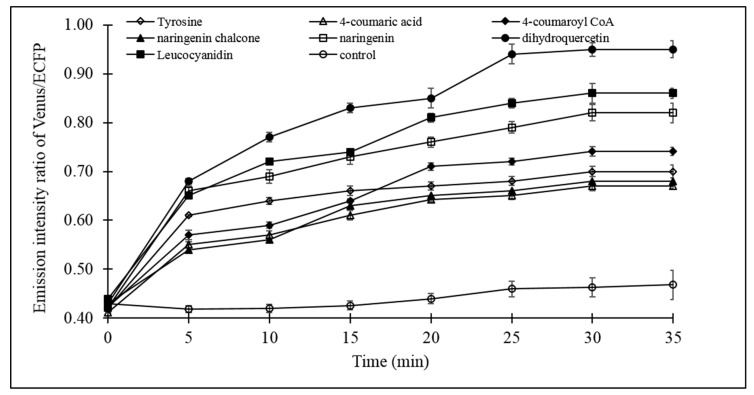
Live cell dynamics of (+)-catechin biosynthesis in bacterial cell. Catechin biosynthesis was measured on the basis of the emission intensity ratio of Venus/ECFP (FRET ratio). Values are mean of three independent replicates. Vertical bars show standard error.

The catechin dynamic experiment with live bacterial cells provided detailed insight of the (+)-catechin flux. Fluxomic analysis of the (+)-catechin biosynthesis pathway revealed the effect of the addition of the substrates (tyrosine, 4-coumaric acid, 4-coumaroyl CoA, naringeninchalcone, naringenin, dihydroquercetin, leucocyanidin) individually on (+)-catechin flux. Microplate analysis showed significant increase in the emission intensity ratio of Venus/ECFP upon addition of all the substrates, relative to the control, which was devoid of any of the substrates of the (+)-catechin biosynthesis pathway. The increase in the emission intensity ratio of Venus/ECFP, observed only after the addition of substrates of (+)-catechin biosynthesis pathway, confirms the efficient capability of FLIP-Cat in screening the (+)-catechin flux in real time. The data obtained through microplate analysis clearly revealed a slow increase in the emission intensity ratio of Venus/ECFP in the first 5 min after the addition of tyrosine, 4-coumaric acid, 4-coumaroyl CoA, naringenin chalcone, naringenin, dihydroquercetin, and leucocyanidin (Figure 6). Thereafter, variation in the increase in the FRET ratio was reported with different substrates. In the case of tyrosine (PAL), 4-coumaric acid (4 CL), and naringenin chalcone (CHI), a gradual increase in the emission intensity ratio of Venus/ECFP was observed until 20 min, followed by a minimal rise, indicating saturation. Almost the same pattern of increase in the emission intensity ratio of Venus/ECFP was observed for 4-coumaroyl CoA (CHS), which increased slightly until 10 min, followed by a sharp increase until 20 min and a slight increase until 30 min, moving ahead to saturation. For naringenin, which is the substrate for F3H, a gradual increase was observed until 30 min, followed by saturation. However, for leucocyanidin, it was observed that after the increase in the emission intensity ratio of Venus/ECFP until 10 min, there was slight decline at 15 min, which was again followed by a gradual increasing until 30 min, before saturation. However, with the addition of dihydroquercetin, which is the substrate for the enzyme DFR, a remarkable increase in the emission intensity ratio of Venus/ECFP was observed relative to all the other substrates, reaching its maximum at 25 min, followed by the saturation. As mentioned earlier, the emission intensity ratio of Venus/ECFP corresponds to the flux of (+)-catechin. Therefore, a higher change in the ratio corresponds to a higher concentration measured using the FLIP-Cat sensor. Thus, the possible role of DFR in the regulation of (+)-catechin production can be inferred through the results.

#### 3.4.2. Confocal Analysis

**Figure 7:**
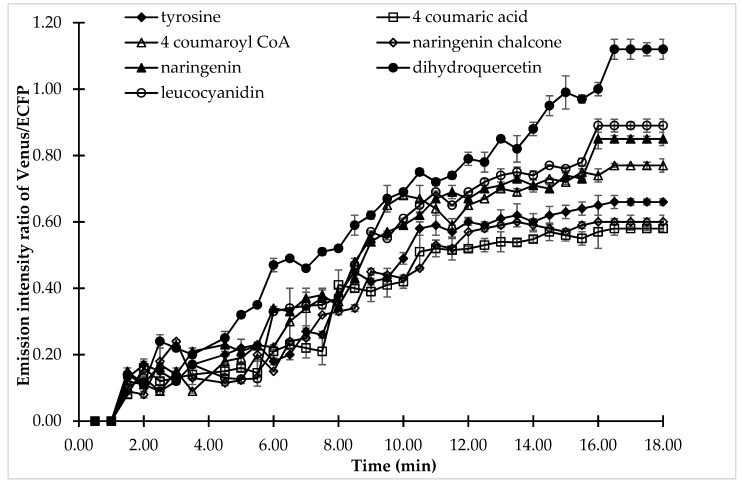
Live cell dynamics of (+)-catechin production in the same bacterial cell upon addition of the individual substrates of the (+)-catechin biosynthesis pathway. The graph represents the results of confocal analysis of the same cell, which was examined for (+)-catechin production by adding the substrates of (+)-catechin biosynthesis. (+)-Catechin production is measured on the basis of the emission intensity ratio of Venus/ECFP. Values plotted are means of three independent replicates. Vertical bar indicates standard error.

Confocal analysis was carried out to understand the dynamics of (+)-catechin flux in single cells. Confocal analysis gave more precise information about the (+)-catechin flux regulation by analyzing the effect of all the substrates of the (+)-catechin biosynthesis pathway on a single cell exposed to the same physical or environmental perturbation (Figure 7). 4-coumaric acid, the substrate for 4CL, showed the least effect on FRET ratio changes, while dihydroquercetin, which is the substrate for DFR, showed the maximum increase in FRET, thereby pronouncing its effect on (+)-catechin flux regulation. A change in the FRET ratio corresponding to catechin production was observed after 2 min followed by perfusion of the bacterial cell with the substrates of the (+)-catechin biosynthesis pathway. However, significant variation was observed in the FRET ratio in the case of different substrates after 2 min, reflecting variation in the (+)-catechin flux. A gradual increase in the emission intensity ratio of Venus/ECFP was observed in the case of 4-coumaric acid, followed by naringenin chalcone, and tyrosine, which reached its maximum (0.58, 0.6, and 0.66, respectively) at 16.5 min and then saturated. Tyrosine was followed by 4-coumaroyl CoA, the substrate for CHS, which showed a maximum FRET ratio of 0.77 at 16.5 min, followed by its saturation. This indicates 4-coumaroyl CoA is more important for (+)-catechin flux then tyrosine, which in turn has more effect than naringenin chalcone, which is again more superior than 4-coumaric acid.

However, the last three substrates of the (+)-catechin biosynthesis pathway are more important when compared with the starting substrates. It was observed that when cells were perfused with naringenin and leucocyanidin, the emission intensity ratio reached 0.85 and 0.89, respectively followed by saturation. When the cells were perfused with the DFR (dihydroquercetin), a significant and large change was observed in the emission intensity ratio of Venus/ECFP, which reached maximum at 16.5 min, showing a FRET ratio of 1.12. This increase in the emission intensity ratio Venus/ECFP after the perfusion of cells with the DFR substrate dihydroquercetin suggested the possible role of DFR in (+)-catechin flux regulation. The pattern of substrate effect on the regulation of (+)-catechin flux followed the same pattern as was observed in microplate analysis.

## 4. Discussion

The present study showed successful development of a FRET-based nanosensor for real-time monitoring of the flux of (+)-catechin in bacterial cells. The success of the nanosensor depends upon the ligand binding element and the fluorophore pair. For FRET to occur, there is a pre-requisite of having donor and acceptor fluorophore close to each other at a distance of >10 nm, which is accomplished by employing ligand-binding proteins. Hence, the ligand-binding element must be capable of undergoing conformational change in the presence of its ligand, which will result in bringing the fluorophore pair closer to each other. In the present study, fraa-3 from *Fragaria ananassa* was chosen as the (+)-catechin binding element. (+)-Catechin on binding with fraa-3 forms a closed structure by bringing sufficient conformational structural change in its open structure [27]. This shift in structure from open to closed form of fraa-3 is sufficient enough to bring the donor and acceptor fluorophore close to each other, thus resulting in a fluorescence resonance energy transfer (FRET) phenomenon to occur between the fluorophore pair, which is measured as change in the ratio of emission intensities of the donor and acceptor fluorophore. The developed sensor was named FLIP-Cat. The FLIP-Cat has been shown to work in versatile pH making it suitable for in vivo analysis (Figure 2). High specificity of FLIP-Cat towards (+)-catechin makes it an excellent tool for monitoring (+)-catechin in a complex metabolite pool having diverse homologues metabolites (Figure 3). The study confirms the successful expression of the nanosensor protein in a bacterial cell, and the observed change in the emission ratio of the donor and acceptor fluorophore in presence of catechin validated the successful working of the FLIP-Cat. The main aim of developing the nanosensor was to find out the regulatory switch of the (+)-catechin biosynthetic pathway by monitoring the dynamics of (+)-catechin production with high spatial and temporal resolution.

To find out the regulatory switch of the (+)-catechin biosynthetic pathway, the whole pathway of (+)-catechin biosynthesis was introduced into *E. coli*. The pathway proposed by Hwang et al. [16] and Park et al. [15] for (+)-catechin biosynthesis was taken into consideration in the present study. The (+)-catechin biosynthesis pathway involves enzymes PAL, C4H, 4CL, CHS, CHI, F3H, DFR, and LCR. The very first step of the pathway is the deamination of phenylalanine by phenylalanine ammonia-lyase (PAL) to cinnamic acid, which is further hydroxylated to 4-coumaric acid by cinnamate 4-hydroxylase (C4H). Moving to next step, 4-coumaric acid is then activated by enzyme 4-coumaroyl CoA ligase (4CL) to 4-coumaroyl CoA, which is further converted to naringenin chalcone by enzyme chalcone synthase (CHS). Naringenin chalcone then acts as substrate for the enzyme chalcone isomerase (CHI) for the formation of naringenin, which is further stereospecifically hydroxylated to dihydroflavonol by flavanone 3-hydroxylase (F3H). Enzyme DFR reduces the dihydroflavonol (dihydroquercetin) stereospecifcially to leucoanthocyanin (leucocyanindin) which is utilized by the enzyme LCR as substrate to produce (+)-catechin. Employing the approach of metabolic engineering, the whole pathway was introduced into *E. coli* in two sets of plasmids. The present study successfully validated the production of catechin in the recombinant *E. coli* by employing genes involved in the (+)-catechin biosynthetic pathway. These genes, on expression, led to the synthesis of corresponding functional enzymes, which converts the tyrosine to (+)-catechin by going through a chain of reaction. There was hindrance in the production of (+)-catechin in *E. coli* because of the instability of one of the enzymes of the (+)-catechin biosynthetic pathway (C4H). The C4H is membrane-bound cytochrome P-450 hydroxylase which needs molecular oxygen and a reducing equivalentto be expressed functionally. This reducing equivalent is obtained from NADPH (Nicotineamide adenine dinucleotide phosphate hydrogen) and delivered to C4H via a specific cytochrome P-450 reductase [31,32], which is absent in bacteria, as a result of which C4H is not successfully expressed in bacteria. Thus, to avoid this difficulty, the PAL enzyme from yeast was used which utilizes tyrosine as its substrate and leads to the formation of 4-coumaric acid, which is then directly acted on by 4CL enzyme, by passing the step catalyzed by C4H. Addition of promoter and ribosome binding sites in front of each gene improved the gene expression efficiency and protein production respectively [28]. (+)-Catechin and naringenin production using the developed construct was validated using HPLC analysis. HPLC analysis was used only for the validation of the produced compound as it involves invasive and sample disruptive procedures which limit its application in fluxomic analysis.

The other challenge in fluxomic analysis is that the output of the same cell subjected to different perturbations cannot be observed because a different group of cells are analysed in HPLC for every treatment. To understand the regulation of flux of any metabolite, it is mandatory to analyse the response of the same cell under different conditions to precisely understand the regulatory factors or steps. One more disadvantage of HPLC is that real-time monitoring of the metabolite level cannot be carried out. For fluxomics study, the response of cells towards metabolite production in real time is necessary through detection of metabolite production in an engineered microorganism, which will aid in understanding biosynthesis pathways more accurately by monitoring the effect of each step on metabolite production. The present study has offered a potential tool by developing a FRET-based genetically-encoded FLIP-Cat sensor that is non-invasive and has the capability to monitor (+)-catechin flux in real time in a single cell and thus help in investigating metabolic flux of (+)-catechin biosynthesis.

After successfully validating the naringenin and catechin production using the plasmid pET26b-PT7-rbs-PAL-PT7-rbs-4CL-PT7-rbs-CHS-PT7-rbs-CHI and pET26b-PT7-rbs-F3H-PT7-rbs-DFR-PT7-rbs-LCR, fluxomic analysis of (+)-catechin was carried out using the FLIP-Cat sensor. The effect of each substrate on (+)-catechin flux was successfully screened using the developed FLIP-Cat sensor. Monitoring of (+)-catechin flux was based on the measurement of the FRET ratio (emission intensity ratio of Venus/ECFP). Initially, a group of cells were used to understand the flux of (+)-catechin using a microplate reader. Data obtained through microplate analysis suggested addition of 4-coumaric acid (4CL) exhibited the least increase in FRET ratio when compared with the other substrates. After 4-coumaric acid, naringenin chalcone was found to have less role in (+)-catechin flux regulation, as the latter also showed only a minimal increase in the FRET ratio, followed by tyrosine (PAL), 4-coumaroyl CoA (CHS), naringenin (F3H), leucocyanidin (LCR), and dihydroquercetin (DFR).

In the case of leucocyanidin, a slight decrease was observed in the FRET ratio, followed by a gradual increase. However, the increase was further resumed after just 5 min. The maximum and a sharp increase in the FRET ratio was obtained only after the addition of dihydroquercetin, which is the substrate for the enzyme DFR, suggesting the important role of DFR. After microplate analysis, confocal analysis was performed to understand the flux of (+)-catechin in a single cell. In confocal analysis, the response of the same cell was monitored with respect to different substrates. This in turn, provided a more precise and accurate knowledge of the (+)-catechin biosynthesis pathway. In confocal analysis, after perfusing the *E. coli* cell with the substrates for 2 min, an increase in the emission intensity ratio of Venus/ECFP was observed, indicating that (+)-catechin production started after a perfusion of 2 min, followed by increase in the ratio differentially for different substrates and then reaching a point called saturation at 16.5 min. As mentioned earlier, change in the emission intensity ratio of Venus/ECFP is a measure of catechin flux, the effect of substrates on catechin flux can be easily inferred. The substrate that has the least role in (+)-catechin flux regulation was found to be 4-coumaric acid, as it showed the least increase in the emission intensity ratio of Venus/ECFP (0.58), while the substrate that demonstrated a significantly important effect on (+)-catechin flux was dihydroquercetin, as it showed an increase of 1.12 in the emission intensity ratio of Venus/ECFP, which was more than the increase of any of the other substrates. 4-coumaric acid, naringenin chalcone, tyrosine, and 4-coumaroyl CoA were found to have slightly less significant role in (+)-catechin flux regulation, as they showed minimal increase in the emission intensity ratio of Venus/ECFP, which was0.6, 0.66, and 0.77, respectively. Although the increase in ratio for the substrate naringenin (0.85) and leucocyanidin (0.89) were larger than the naringenin chalcone, tyrosine, 4-coumaroyl CoA, and 4 coumaric acid, it was not large enough to decide their regulatory role in the flux of (+)-catechin. Flux analysis of (+)-catechin biosynthesis by the addition of different substrates suggested the importance of DFR in the regulation of (+)-catechin flux. Specificity analysis showed that the FLIP-Cat did not sense the dihydroquecetin and leucocyanidin, conferring that the high FRET change upon addition of dihydroquercetin was due to the change in catechin flux corresponding to its maximum production. The order of the regulatory effect of the substrates on catechin flux was found to be dihydroquecetin (DFR) > leucocyanidin (LCR) > naringenin(F3H) > 4-coumaroyl CoA (CHS) > tyrosine (PAL)> naringenin chalcone (CHI) > 4-coumaric acid (4CL). It is suggested that the DFR could be the regulatory element of the (+)-catechin biosynthesis pathway. This study is in synchrony with the previous results where it was shown that it is necessary to improve the DFR requirement in order to enhance (+)-catechin production [10,30]. Chemler et al. [10] have shown that by limiting the supply of oxygen, the production of (+)-catechin can be enhanced as oxygen availability acts as a limiting factor for DFR [33].

## 5. Conclusions

In this study, a genetically-encoded FRET-based nanosensor (FLIP-Cat) was successfully developed for real-time monitoring of the flux of (+)-catechin in living cells. The sensor was stable and very specific to the (+)-catechin. Later, a (+)-catechin biosynthesis pathway was designed and constructed in two plasmids. The pathway was introduced in *E. coli* along with the FLIP-Cat. Metabolic flux of the (+)-catechin was monitored in real time in the bacterial cells after adding different substrates of the (+)-catechin biosynthesis pathway. It is suggested that DFR could be the regulatory element of the catechin biosynthesis pathway. Information regarding this regulatory element of the (+)-catechin biosynthesis pathway can be used for manipulating the (+)-catechin biosynthesis pathway using a metabolic engineering approach to enhance production of catechin.

## Supplementary Materials

The supplementary materials are available online at https://www.mdpi.com/2076-3921/9/4/288/s1, Figure S1. Catechin biosynthetic pathway in plants adopted from Hwang et al. (2003) and Park et al. (2004)**;** Figure S2. Schematic representation of pGEMT-Easy_ECFP_Fraa-3_Venus; Figure S3. Schematic representation of pRSETB_ECFP_Fraa3_Venus; Figure S4. Schematic depiction of the construct pET26b-PT7-rbs-PAL-PT7-rbs-4CL-PT7-rbs-CHS-PT7-rbs-CHI; Figure S5. Schematic depiction of the construct pET26b-PT7-rbs-F3H-PT7-rbs-DFR-PT7-rbs-LCR; Figure S6. Designing of nanosensor. Figure S7. Nucleotide sequences of the cloned fraa-3, ECFP and Venus; Figure S8. PCR amplified fraa-3 gene of band size 500bp; Figure S9: Recombinant construct of pGEMT-Easy_ECFP_Fraa-3_Venus; Figure S10. Restriction digestion of pRSET-B_ECFP_Fraa-3 _Venus with *Age* I and *Bst*E II yielding two band size of 4.5 kb and 0.5 kb; Figure S11. Recombinant construct of pRSET-B_ECFP_Fraa-3_Venus of band size 4.8 kb; Figure S12. Restriction digestion of pRSET-B_ECFP_Fraa-3_Venus with *Age* I and *Bst*EII yielding two band size of 4.3 kb and 0.5 kb; Figure S13. HPLC chromatogram of standard catechin showing RT at 6.013 min; Figure S14. HPLC chromatogram of standard naringenin showing RT at 14 min. Table S1. Primers for amplification of Fraa-3, ECFP and Venus genes; Table S2. Primers for amplification for various genes.

## References

[1] Lobo V., Patil A., Phatak A., Chandra N. (2010). Free radicals, antioxidants and functional foods: Impact on human health. Pharmacogn. Rev..

[2] Li S., Chen G., Zhang C., Wu M., Wu S., Liu Q. (2014). Research progress of natural antioxidants in foods for the treatment of diseases. Food Sci. Human Wellness..

[3] Mandal S., Yadav S., Yadav S., Nema R.K. (2009). Antioxidants: A review. J. Chem. Pharm..

[4] Kurutas E.B. (2015). The importance of antioxidants which play the role in cellular response against oxidative/nitrosative stress: Current state. Nutri. J..

[5] Khan N., Mukhtar H. (2007). Tea polyphenols for health promotion. Life Sci..

[6] Dufresne C.J., Farnworth E.R. (2001). A review of latest research findings on the health promotion properties of tea. J. Nutr. Biochem..

[7] Higdon J.V., Frei B. (2003). Tea catechins and polyphenols: Health effects, metabolism, and antioxidant functions. Crit. Rev. Food Sci. Nutr..

[8] McKay D.L., Blumberg J.B. (2002). The role of tea in human health: An update. J. Am. Coll. Nut..

[9] Demeule M., Michaud-Levesque J., Annabi B., Gingras D., Boivin D., Lamy S., Bertrand Y., Beliveau R. (2002). Green tea catechins as novel antitumor and antiangiogenic compounds. Curr. Med. Chem..

[10] Chemler J.A., Lock L.T., Koffas M.A., Tzanakakis E.S. (2007). Standardized biosynthesis of flavan-3-ols with effects on pancreatic beta-cell insulin secretion. Appl. Microbiol. Biotechnol..

[11] Manikandan R., Beulaja M., Arulvasu C., Sellamuthu S., Dinesh D., Prabhu D., Babu G., Vaseeharan B., Prabhu N.M. (2012). Synergistic anticancer activity of curcumin and catechin: An in vitro study using human cancer cell lines. Microsc. Res. Tech..

[12] Yamamoto M., Nakatsuka S., Otani H., Kohmoto K., Nishimura S. (2000). (+)-Catechin acts as an infection-inhibiting factor in strawberry leaf. Phytopathology.

[13] Kottawa-Arachchi J.D., Gunasekare M.K., Ranatunga M.A. (2019). Biochemical diversity of global tea [*Camellia sinensis* (L.) O. Kuntze] germplasm and its exploitation: A review. Genet Resour Crop Ev..

[14] Lambert J.D., Lee M.J., Lu H., Meng X., Hong J.J., Seril D.N., Sturgill M.G., Yang C.S. (2003). Epigallocatechin-3-gallate is absorbed but extensively glucuronidated following oral administration to mice. Nutr. J..

[15] Park J.S., Kim J.B., Hahn B.S., Kim K.H., Ha S.H., Kim J.B., Kim Y.H. (2004). EST analysis of genes involved in secondary metabolism in *Camellia sinensis* (tea), using suppression subtractive hybridization. Plant. Sci..

[16] Hwang E.I., Kaneko M., Ohnishi Y., Horinouchi S. (2003). Production of plant-specific flavanones by *Escherichia coli* containing an artificial gene cluster. Appl. Environ. Microbiol..

[17] Farr’e G., Blancquaert D., Capell T., Van Der Straeten D., Christou P., Zhu C. (2014). Engineering complex metabolic pathways in plants. Annu. Rev. Plant Biol..

[18] Stephanopoulos G. (1999). Metabolic fluxes and metabolic engineering. Metab. Eng..

[19] Lalonde S., Ehrhardt D.W., Frommer W.B. (2005). Shining light on signaling and metabolic networks by genetically encoded biosensors. Curr. Opin. Plant. Biol..

[20] Ameen S., Ahmad M., Mohsin M., Qureshi M.I., Ibrahim M.M., Abdin M.Z., Ahmad A. (2016). Designing, construction and characterization of genetically encoded FRET-based nanosensor for real time monitoring of lysine flux in living cells. J. Nanobiotechnol..

[21] Besnard J., Okumoto S. (2014). Glutamine flux imaging using genetically encoded sensors. J. Vis. Exp..

[22] Fehr M., Frommer W.B., Lalonde S. (2002). Visualization of maltose uptake in living yeast cells by fluorescent nanosensors. Proc. Natl. Acad. Sci. USA.

[23] Hu H., Gu Y., Xu L., Zou Y., Wang A., Tao R., Chen X., Zhao Y., Yang Y. (2017). A genetically encoded toolkit for tracking live-cell histidine dynamics in space and time. Sci. Rep..

[24] Whitfield J.H., Zhang W.H., Herde M.K., Clifton B.E., Radziejewski J., Janovjak H., Hanneberger C., Jackson C.J. (2015). Construction of a robust and sensitive arginine biosensor through ancestral protein reconstruction. Protein Sci..

[25] Ahmad M., Mohsin M., Iqrar S., Manzoor O., Siddiqi T.O., Ahmad A. (2018). Live cell imaging of vitamin B12 dynamics by genetically encoded fluorescent nanosensor. Sens. Actuator B-Chem..

[26] Sandmann G. (2002). Combinatorial biosynthesis of carotenoids in a heterologous host: A powerful approach for the biosynthesis of novel structures. Chem. Bio. Chem..

[27] Casanal A., Zander U., Munoz C., Dupeux F., Luque I., Botella M.A., Schwab W., Valpuesta V., Marquez J.A. (2013). The strawberry pathogenesis-related 10 (PR-10) Fra a proteins control flavonoid biosynthesis by binding to metabolic intermediates. J. Biol. Chem..

[28] Umar K.M., Abdulkarim S.M., Radu S., Abdul Hamid A., Saari N. (2012). Engineering the production of major catechins by *Escherichia coli* carrying metabolite genes of *Camellia sinensis*. Sci. World J..

[29] Kaneko M., Ohnishi Y., Horinouchi S. (2003). Cinnamate: Coenzyme A ligase from the filamentous bacterium Streptomyces coelicolor A3 (2). J. Bacteriol..

[30] Chemler J.A., Fowler Z.L., McHugh K.P., Koffas M.A. (2010). Improving NADPH availability for natural product biosynthesis in Escherichia coli by metabolic engineering. Metab. Eng..

[31] Hotze M., Schroder G., Schroder J. (1995). Cinnamate 4-hydroxylase from *Catharanthus roseus* and a strategy for the functional expression of plant cytochrome P450 proteins as translational fusions with P450 reductase in Escherichia coli. FEBS Lett..

[32] Pompon D., Louerat B., Bronine A., Urban P. (1996). Yeast expression of animal and plant P450s in optimized redox environments. Methods Enzymol..

[33] Fischer D., Stich K., Britsch L., Grisebach H. (1988). Purification and characterization of (+) dihydroflavonol (3-hydroxyflavanone) 4-reductase from flowers of Dahlia variabilis. Arch. Biochem. Biophys..

