# Supplementary material for: Metabolic Flux Analysis of Catechin Biosynthesis Pathways Using Nanosensor
Source: Antioxidants (Basel). 2020 Mar 31;9(4):288. doi: 10.3390/antiox9040288 (PMC7222200; doi:10.3390/antiox9040288)
Supplement: Supplementary file 1 [file antioxidants-09-00288-s001.pdf]

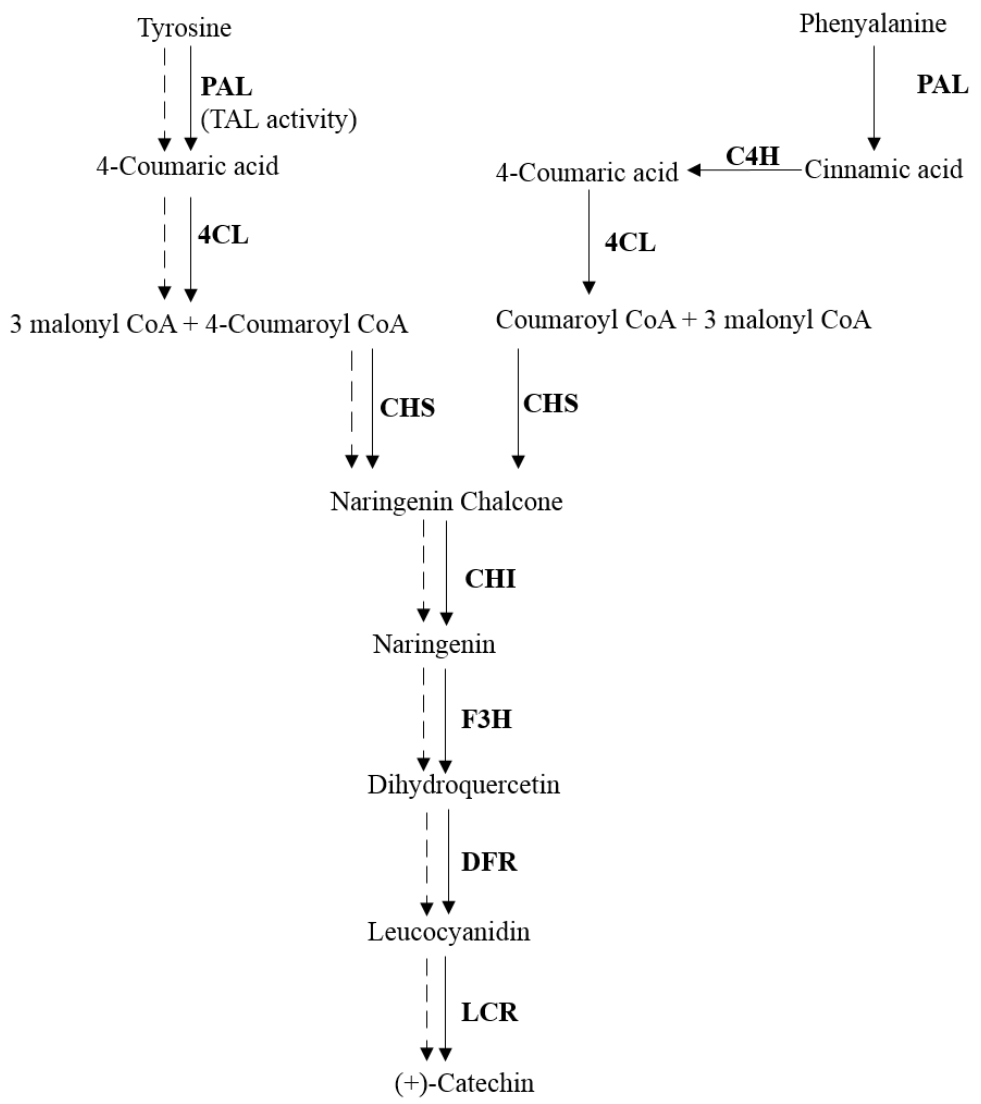

Fig. S1. (+)-Catechin biosynthetic pathway in plants adopted from Hwang et al. (2003) and Park et al. (2004). Dashed arrows represent the engineered catechin pathway in *E. Coli.* PAL : Phenyl - ammonia lyase ; C4H : Cinnamate 4 hydroxylase ; 4CL : 4 Coumarate CoA ligase ; CHS : Chalcone synthase ; CHI : Chalcone isomerase; F3H : Flavanone 3 hydroxylase ; DFR : Dihydroflavonol reductase ; LCR : Leucoanthocyanidin reductase; TAL : Tyrosine-ammonia lyase.

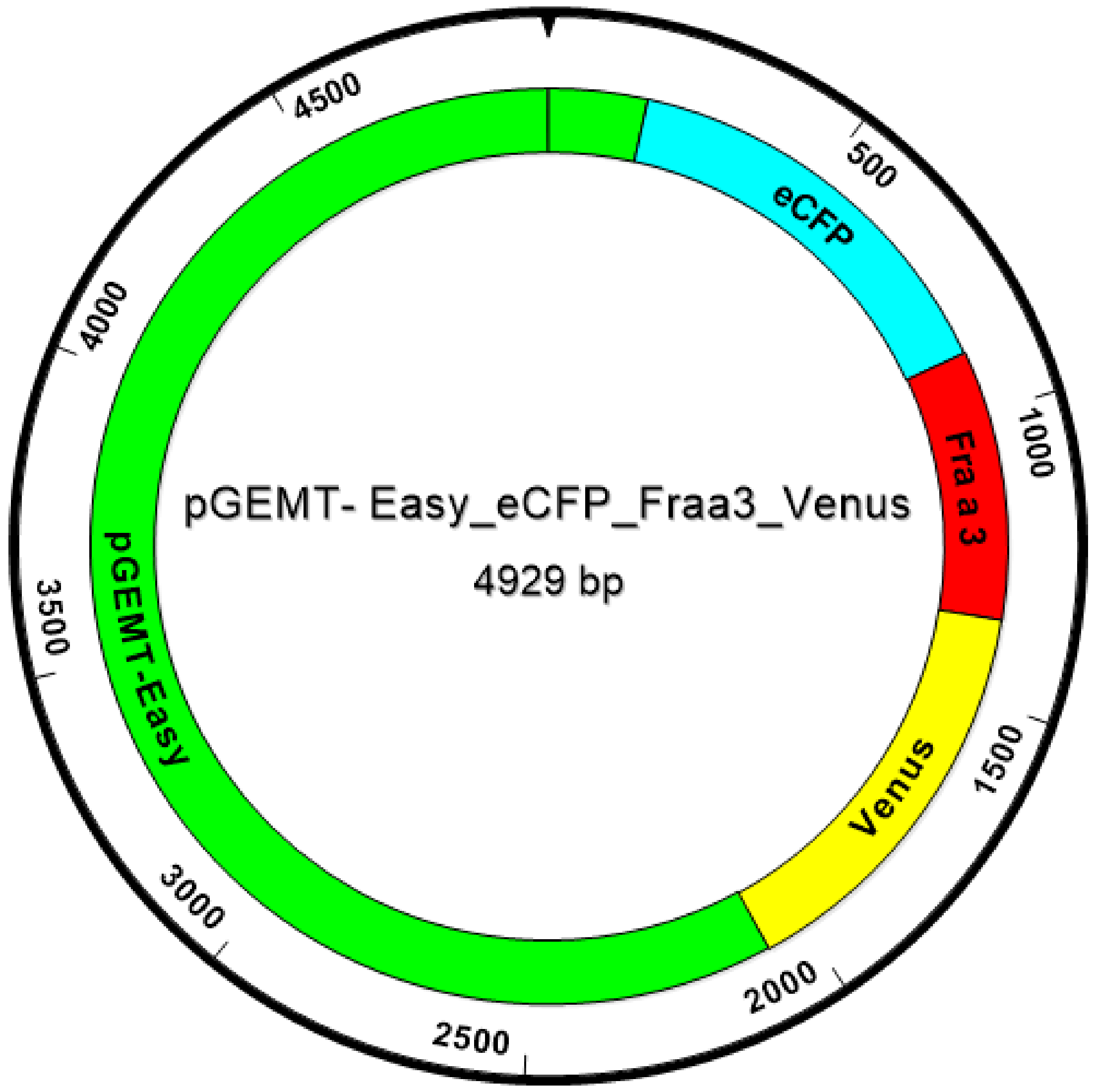

Fig. S2. Schematic representation of pGEMT-Easy_ECFP_Fra a 3_Venus

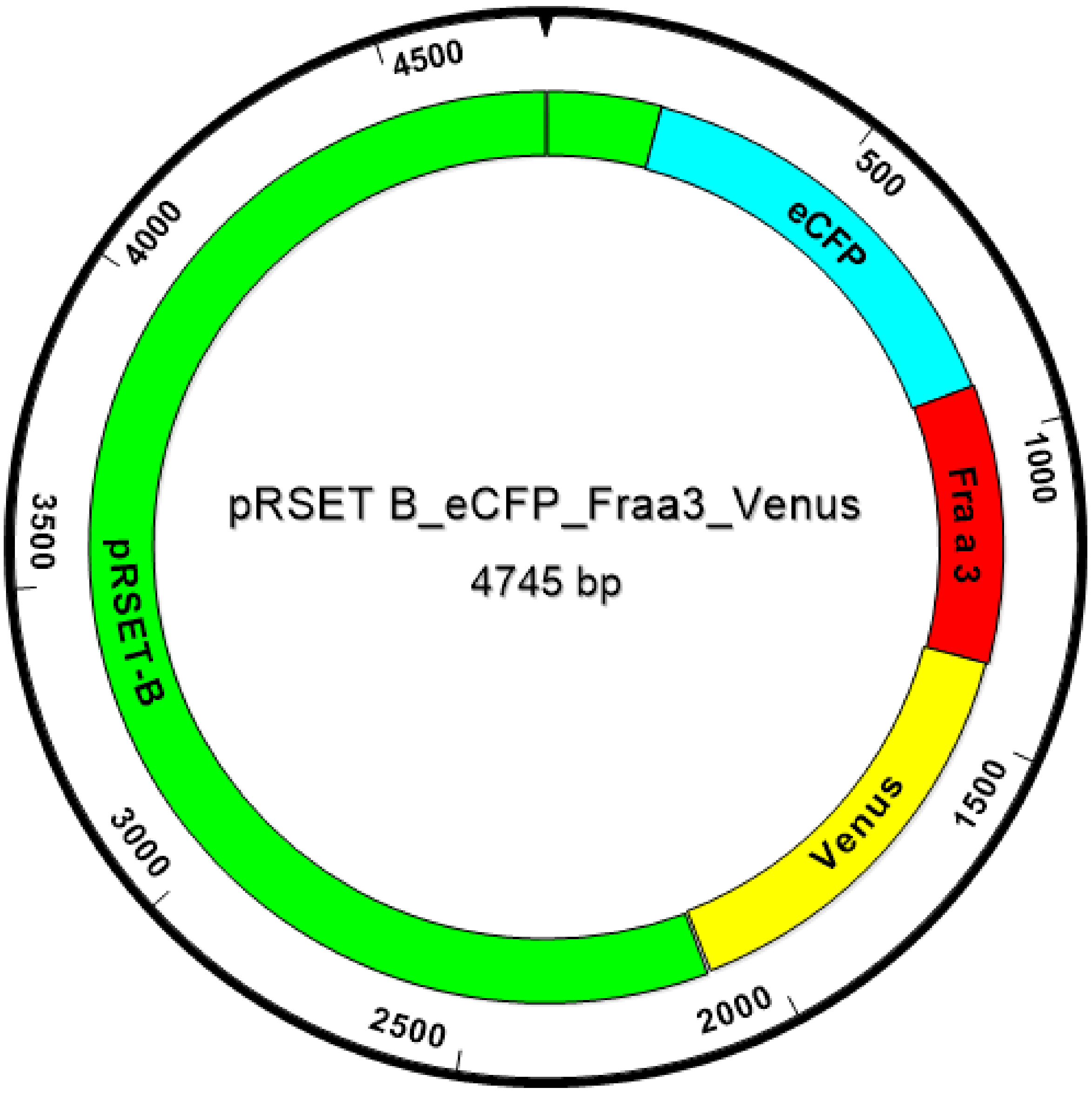

Fig. S3. Schematic representation of pRSETB_ECFP_Fra a 3_Venus

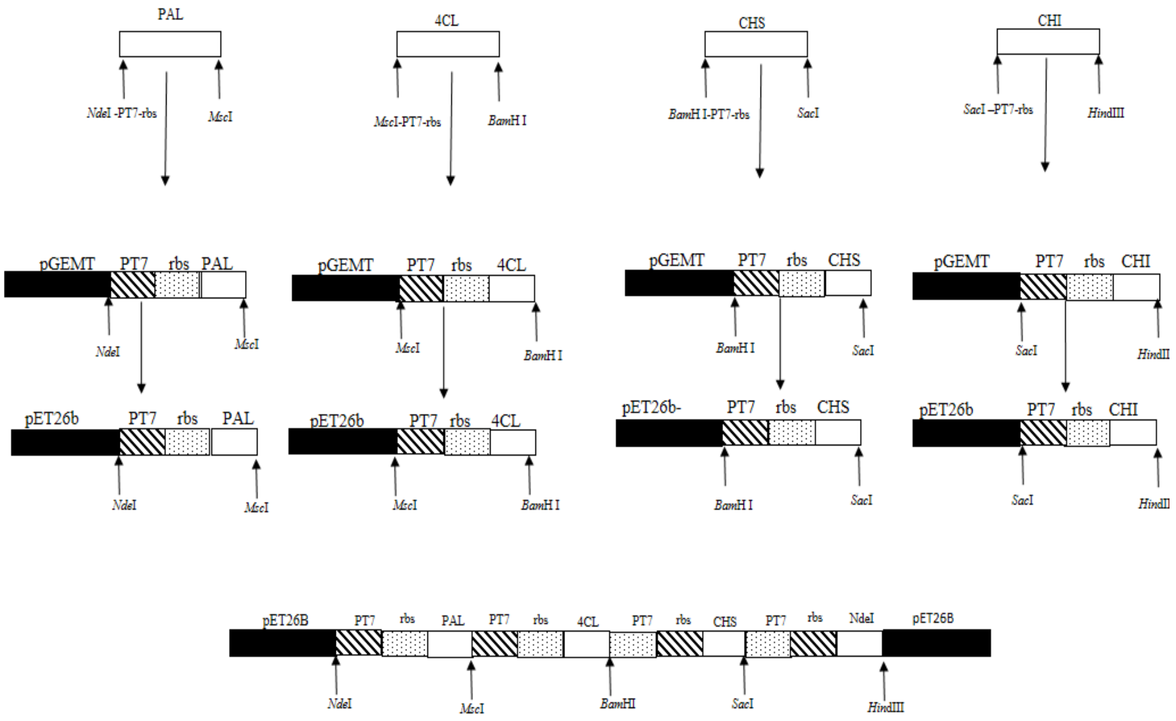

Fig. S4. Schematic depiction of the construct pET26b-PT7-rbs-PAL-PT7-rbs-4CL-PT7-rbs-CHS-PT7-rbs-CHI. Utilising DNA manipulation techniques, PAL, 4CL, CHS and CHI are placed separately under the control of T7 promoter and ribosome binding site in pET26b vector. PAL: Phenyl – ammonia lyase , 4CL : 4 Coumarate CoA ligase, CHS : Chalcone synthase, CHI : Chalcone isomerase, PT7 : Promoter T7, rbs : Ribosome binding site.

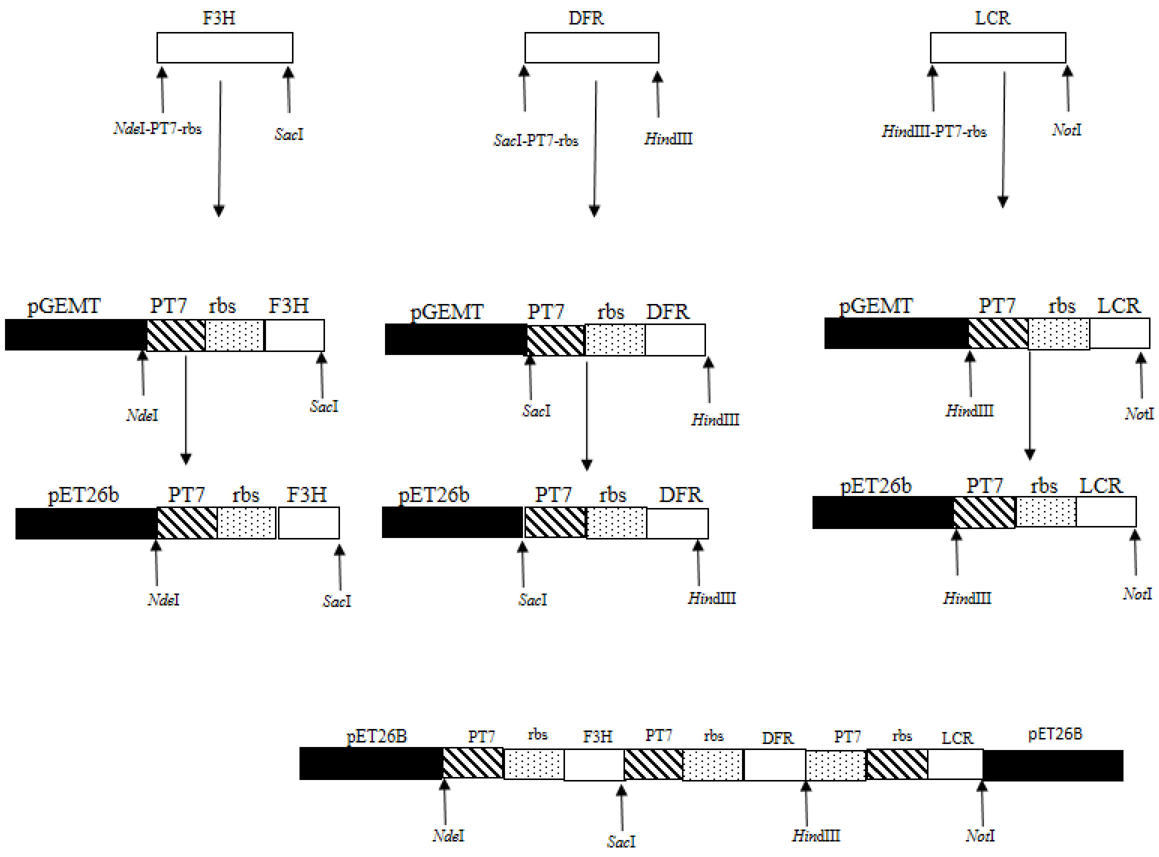

Fig. S5. Schematic representation of the construct pET26b-PT7-rbs-F3H-PT7-rbs-DFR-PT7-rbs-LCR. Employing the earlier DNA manipulation technique, F3H, DFR and LCR are placed separately under the control of T7 promoter and ribosome binding site in pET26 b vector. F3H : Flavanone 3 hydroxylase ; DFR : Dihydroflavolo reductase ; LCR : Leucoanthocyanidin reductase ; PT7 : Promoter T7 ; rbs : Ribosome binding site.

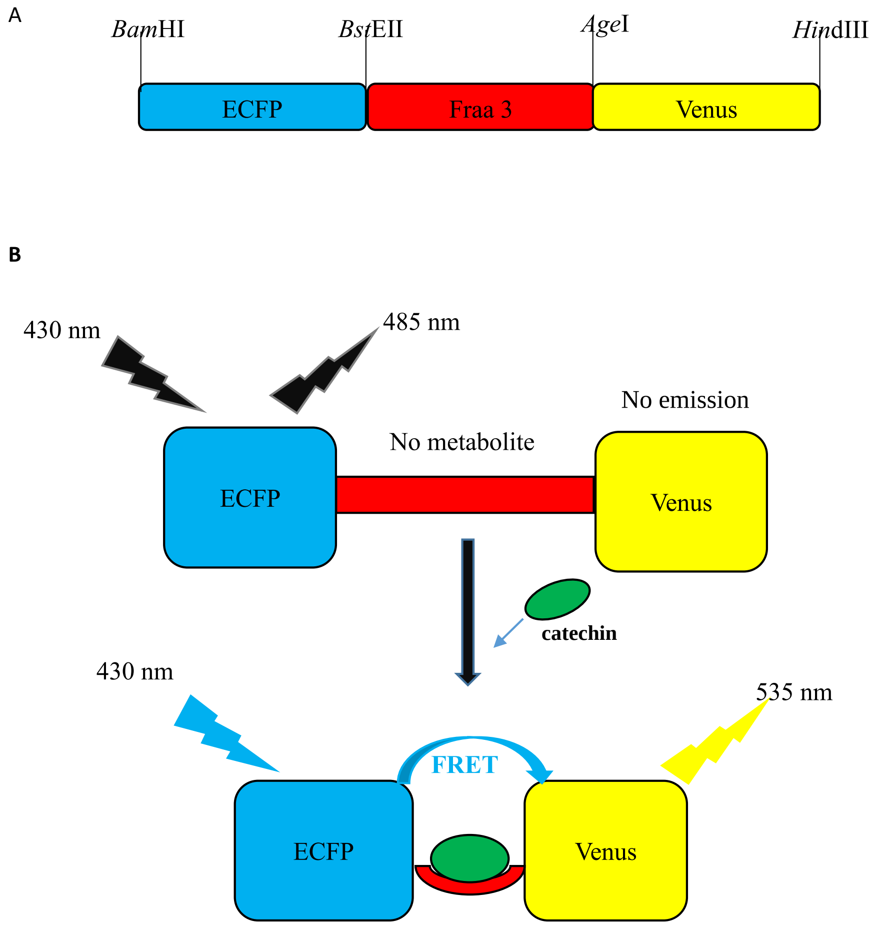

Figure S6. Designing of nanosensor. A. Schematic representation of positions of ECFP, Fraa3, Venus and various restriction endonuclease sits. B. Schematic representation of working of nanosensor. In the absence of (+)-catechin, no conformation in the fraa 3 occurred. Therefore, emission intensity of ECFP could not excite the Venus because of distance <10 nm. By the addition of (+)-catechin, There was conformational change in the fraa 3 that bought ECFP and Venus together at a distance >10 nm. Therefore, emission intensity of the ECFP successfully excites the Venus and causes the FRET.

Nucleotide sequence of Fra a 3

TTCACATACGAATCCGAGTTCACCTCAGTCATCCCACCACCTAAGTTGTTCAAGGCTTTCGTCCTTGACGCCGACAACCTCATCCCCAAGA
TTGCCCCTCAGGCTGTGAAGAGTGCTGAAATCATTGAAGGAGATGGAGGTGTTGGAACCATCAAGAAGATCCATCTTGGTGAAGGAAGT
GAATACAGCTACGTGAAGCATAAGATTGATGGAATCGACAAAGACAACTTCGTGTACAGCTACAGTATCATCGAGGGAGATGCTATCGG
AGACAAGATTGAGAAGATCTCCTATGAGATTAAGTTGGTAGCATCCGGTGGAGGTTCCATCATCAAGAGCACCAGTCACTACCACACCA
AGGGTGAGGTCGAGATTAAGGAAGAGCATGTTAAGGCCGGAAAGGAAAGAGCCGCTGGTCTGTTCAAGATCATTGAGAACCACCTCTTG
GCCCATCCTGAGGAATACAAC

Nucleotide sequence of eCFP

ATGGTGAGCAAGGGCGAGGAGCTGTTCACCGGGGTGGTGCCCATCCTGGTCGAGCTGGACGGCGACGTAAACGGCCACAAGTTCAGCGT
GTCCGGCGAGGGCGAGGGCGATGCCACCTACGGCAAGCTGACCCTGAAGTTCATCTGCACCACCGGCAAGCTGCCCGTGCCCTGGCCCA
CCCTCGTGACCACCCTGACCTGGGGCGTGCAGTGCTTCAGCCGCTACCCCGACCACATGAAGCAGCACGACTTCTTCAAGTCCGCCATGC
CCGAAGGCTACGTCCAGGAGCGCACCATCTTCTTCAAGGACGACGGCAACTACAAGACCCGCGCCGAGGTGAAGTTCGAGGGCGACAC
CCTGGTGAACCGCATCGAGCTGAAGGGCATCGACTTCAAGGAGGACGGCAACATCCTGGGGCACAAGCTGGAGTACAACTACATCAGC
CACAACGTCTATATCACCGCCGACAAGCAGAAGAACGGCATCAAGGCCAACTTCAAGATCCGCCACAACATCGAGGACGGCAGCGTGC
AGCTCGCCGACCA
CTACCAGCAGAACACCCCCATCGGCGACGGCCCCGTGCTGCTGCCCGACAACCACTACCTGAGCACCCAGTCCGCCCTGAGCAAAGACC
CCAACGAGAAGCGCGATCACATGGTCCTGCTGGAGTTCGTGACCGCCGCCGGGATCACTCTCGGCATGGACGAGCTGTACAAG

Nucleotide sequence of Venus

ATGGTGAGCAAGGGCGAGGAGCTGTCACCGGGGTGGTGCCCATCCTGGTCGAGCTGGACGGCGACGTAAACGGCCACAAGTTCAGCGT
GTCCGGCGAGGGCGAGGGCGATGCCACCTACGGCAAGCTGACCCTGAAGCTCATCTGCACCACCGGCAAGCTGCCCGTGCCCTGGCCCA
CCCTCGTGACCACCCTCGGCTACGGCCTCAGTGCTTCGCCCGCTACCCCGACCACATGAAGCAGCACGACTTCTTCAAGTCCGCCATCCC
GAAGGCTACGTCCAGGAGCGCACCATCTTCTTCAAGGACGACGGCAACTACAAGACCCGCGCCGAGGTGAAGTTCGAGGGCGACACCC
TGGTGAACCGCATCGAGCTGAAGGGCATCGACTTAAGGAGGACGGCAACATCCTGGGGCACAAGCTGGAGTACAACTACAACAGCCAC
GACTTCAAGGAGGACGGCAACATCTGGGGCACAAGCTGGAGTACAACTACAACAGCCGCACAACATCGAGGACGGCGGCGTGCAGCTC
GCCGACCACTACCAGCAGAACACCCCCATCGGCGACGGCCCCGTGCTGCTGCCCGACAACCACTACCTGAGCTACCAGTCCAAGCTGAG
CAAGACCCCAACGAGAAGCGCGATCACATGGTCCTGCTGGAGTTCGTGACCGCCGCCGGGATCACTCTCGGCATGGACGAGCTGTACAA

Fig. S7: Nucleotide sequences of the cloned Fra a 3, ECFP and Venus

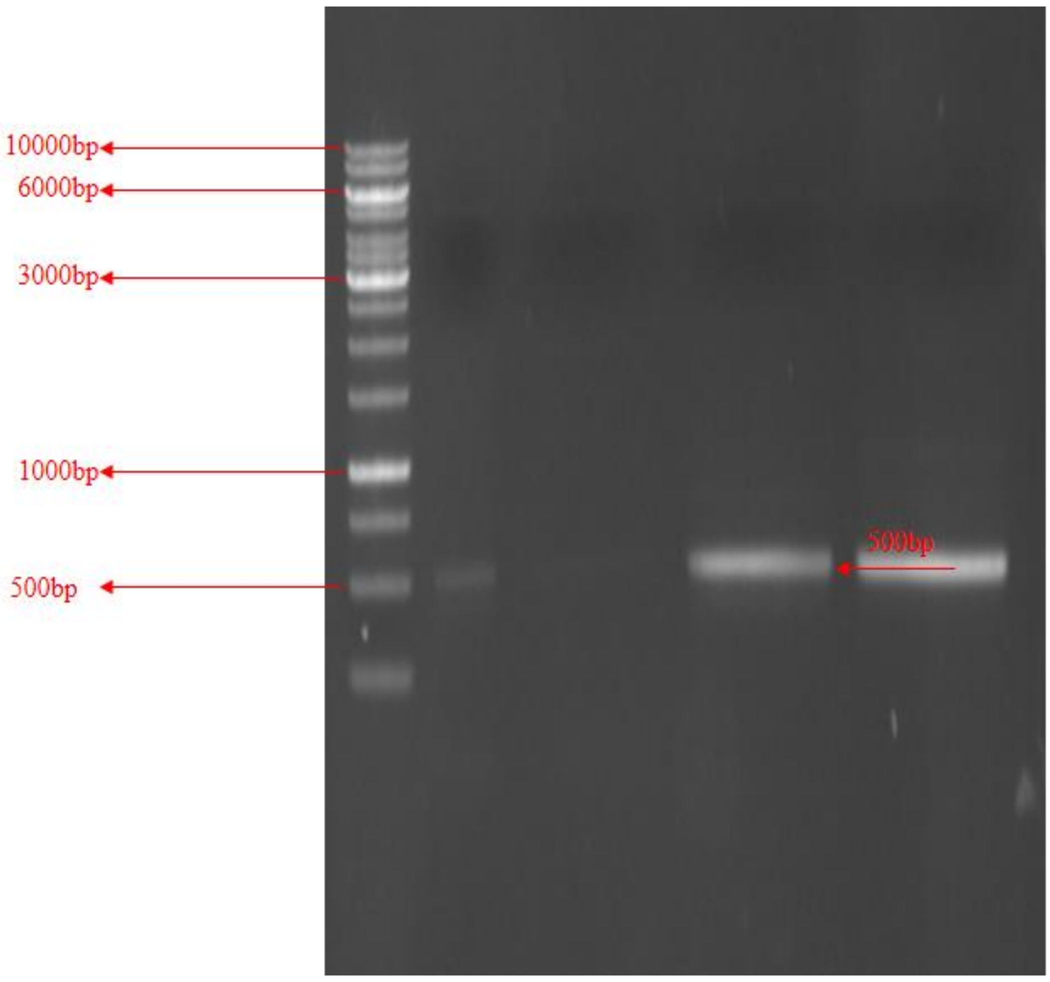

Fig. S8. PCR amplified Fraa 3 gene of band size 500bp

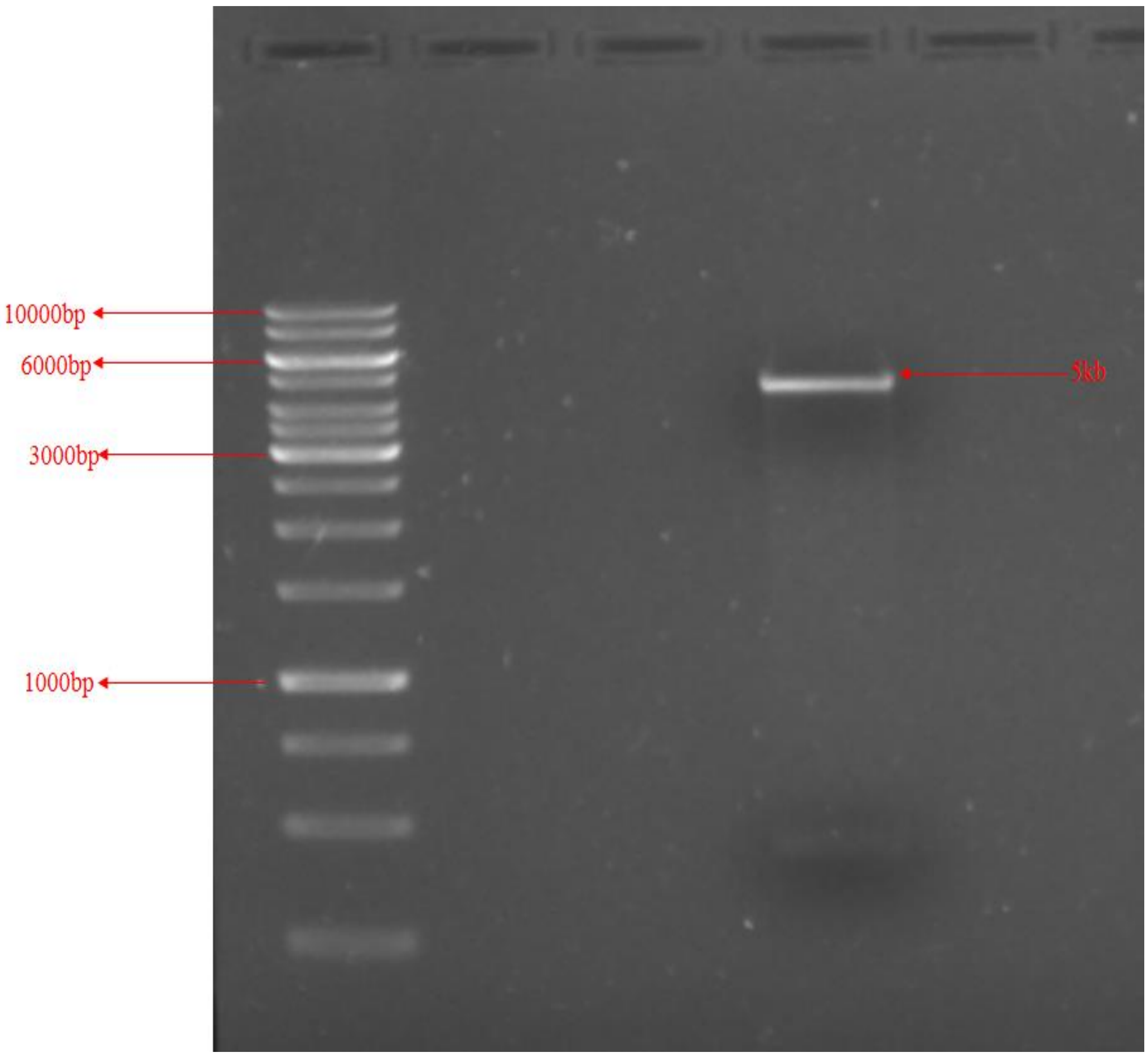

Fig. S9. Recombinant construct of pGEMT-Easy_ECFP_Fra a 3_Venus

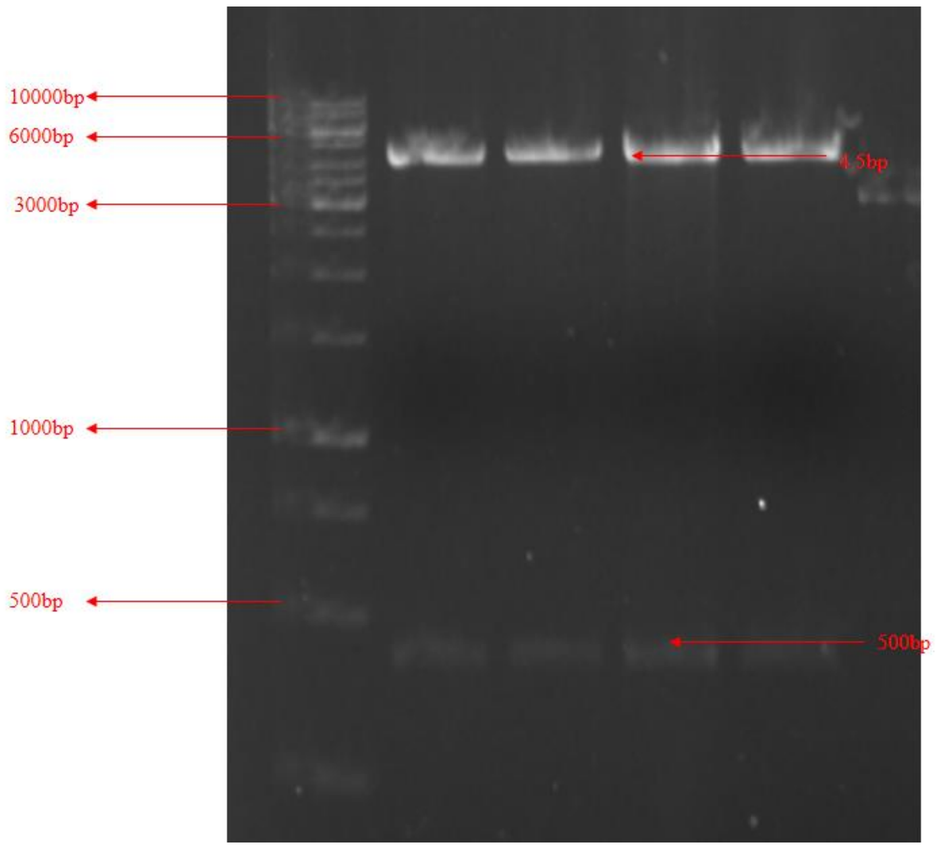

Fig. S10. Restriction digestion of pRSET-B_ECFP_Fra a 3 _Venus with *Age* I and *Bst*E II yielding two band size of 4.5 kb and 0.5 kb

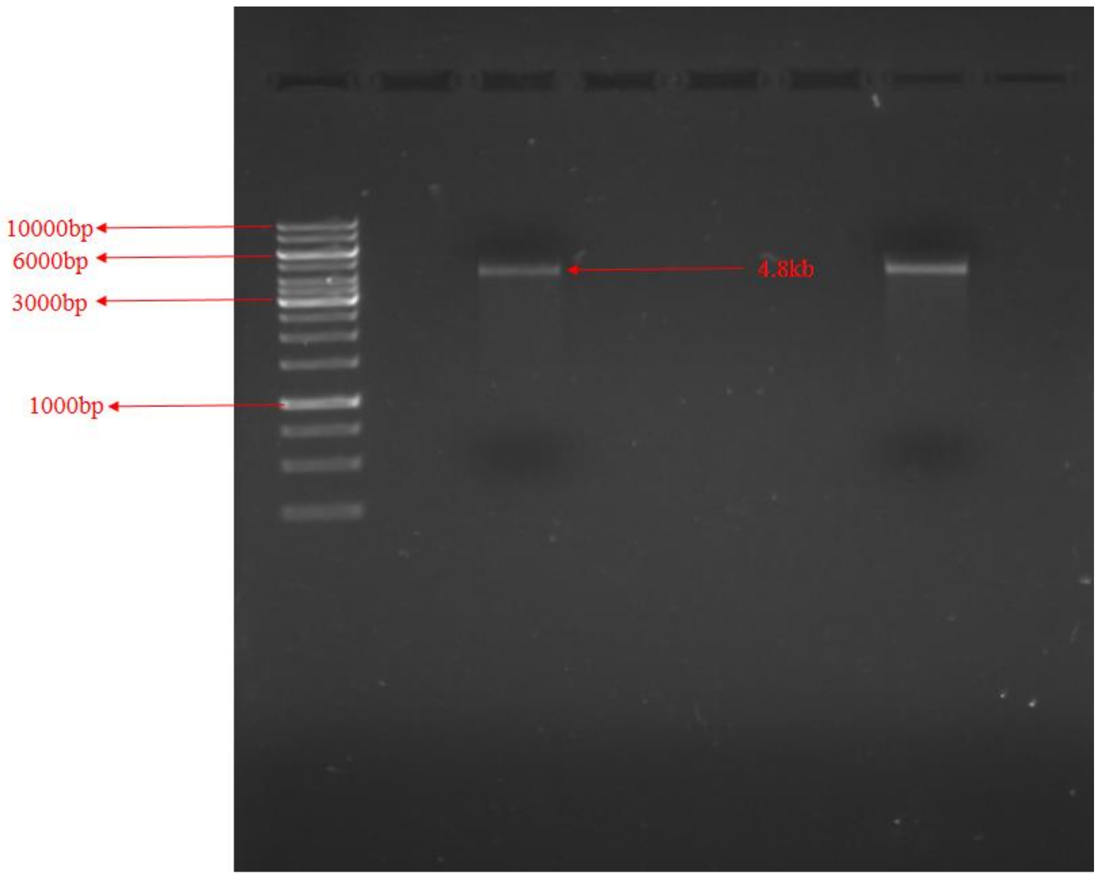

Fig. S11. Recombinant construct of pRSET-B_ECFP_Fra a 3_Venus of band size 4.8 kb

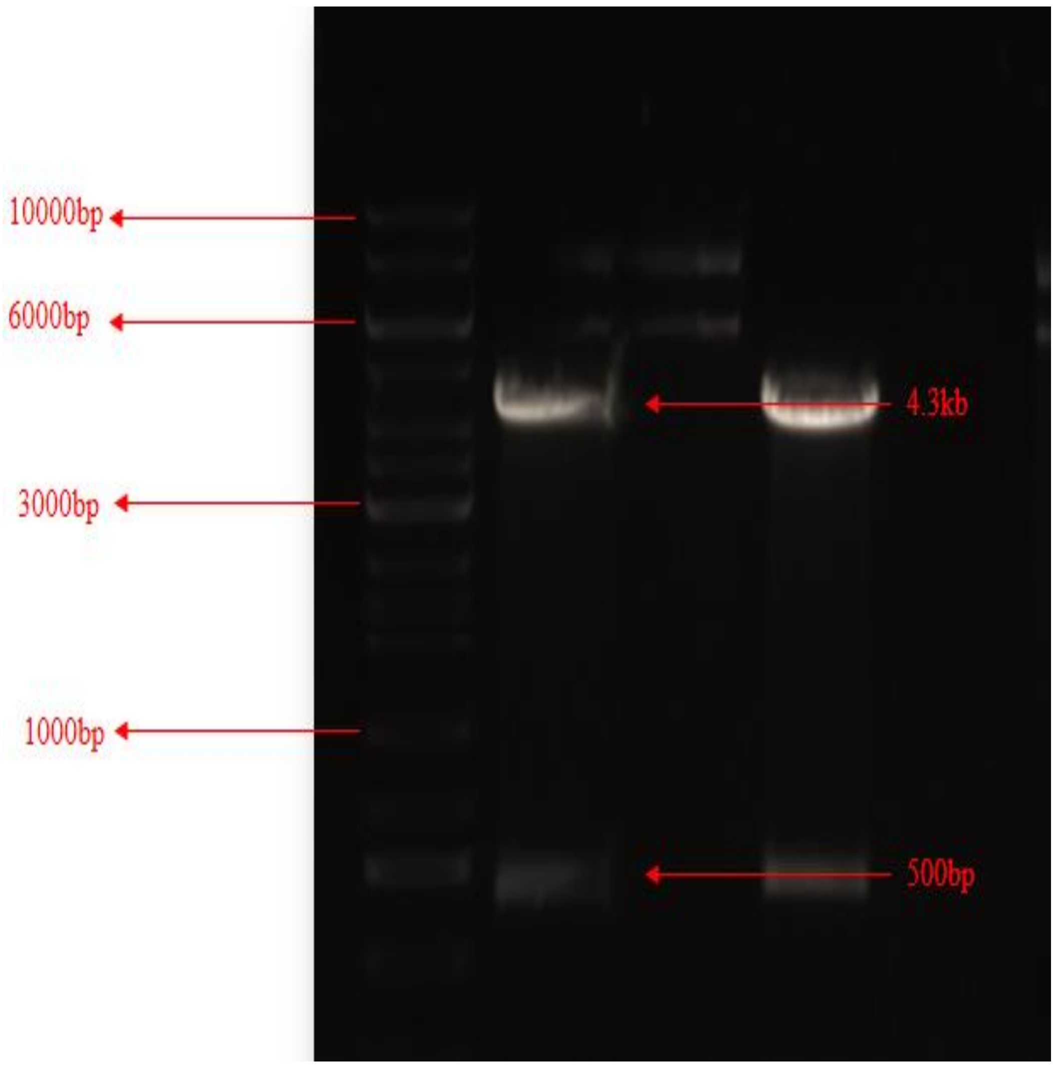

Fig. S12. Restriction digestion of pRSET-B_ECFP_Fra a 3_Venus with *Age* I and *Bst*E II yielding two band size of 4.3 kb and 0.5 kb

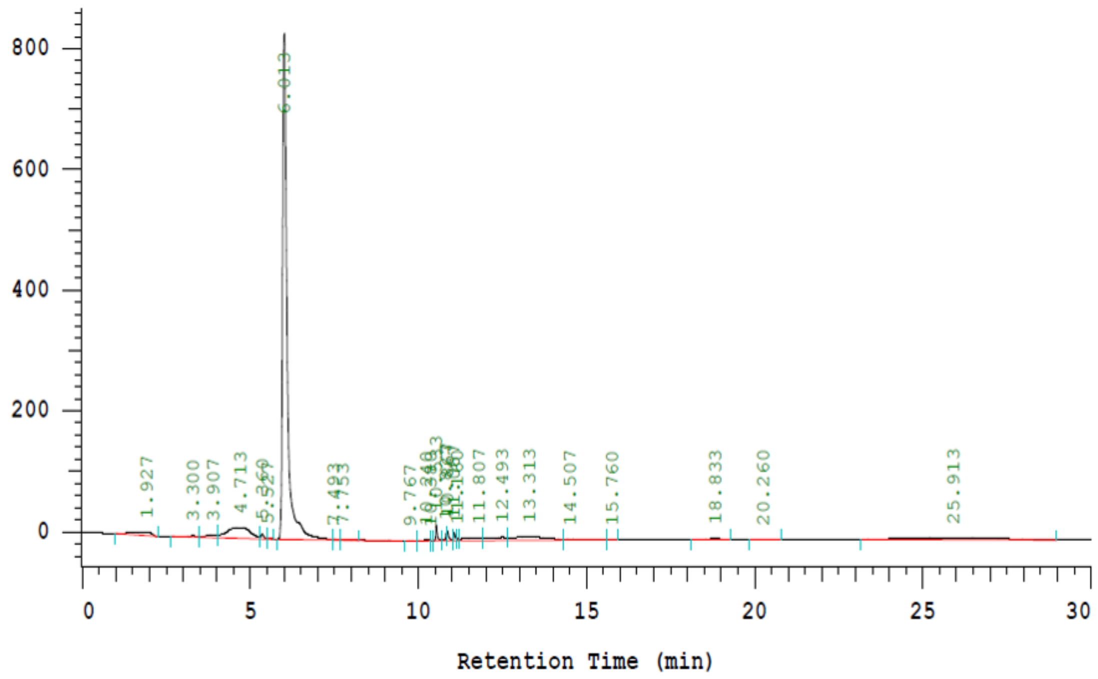

Fig. S13. HPLC Chromatoram of standard catechin showing RT at 6.013 min

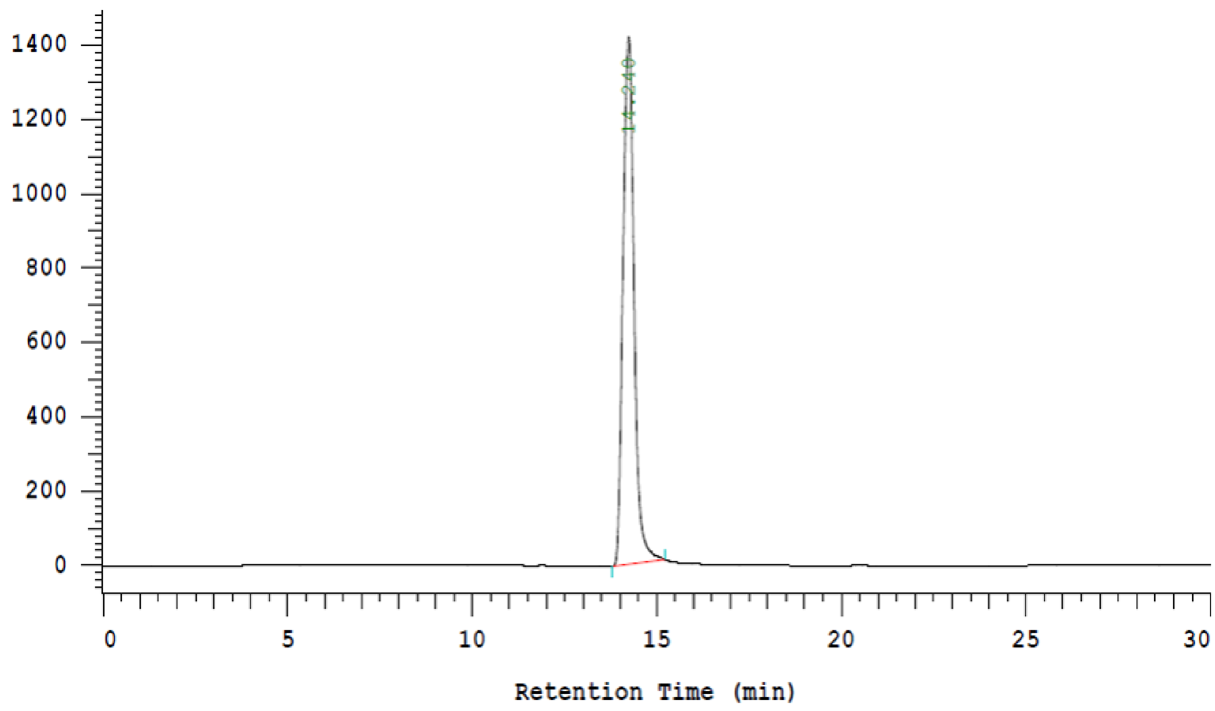

Fig. S14. HPLC Chromatoram of standard naringenin showing RT at 14 min

Table S1. Primers for amplification of Fraa3, ECFP and Venus genes. Bold sequences show the restriction sites

**Fraa 3 primers**
**F.P. with *Bst*EII restriction sites:** 5'- **GGGTTACCC**TTCACATACGAATCCG-3'
**R.P. with *Age*I restriction sites:** 5'- **ACCGGT**GTTGTATTCCTCAGGATGG-3'
**ECFP Primers**
**F.P. with *Bam*HI restriction sites:** 5'- **GGATCC**ATGGTGAGCAAG-3'
**R.P. with *Bst*EII restriction sites:** 5'- **GGGTTACCC**CTTGTACAG CTCGT-3'
**Venus primer**
**F.P. with *Age*I restriction sites:** 5'- **ACCGGT**ATGGTGAGCAAG-3'
**R.P. with *Hin*dIII restriction sites:** 5'- **AAGCTT**TTGTACAGCTCGTCCATGCC-3'

Table S2. Primers for amplification for various genes

*Nde*I-*PT7-rbs-PALF1-F*	5'- CAT ATG **TAATACG** **AAGG** ATG GCC CCC TCC GTC GAC T -3'
*Asc*I-*PALF1-R*	5'- GGC GCG CCG CTC GGA AGT GTA GGC G -3'
*Asc*I-*PT7-rbs-PALF2-F*	5' - GGC GCG CCT **TAATACG** **AAGG** CGG TCA CCT TGC CAA -3'
*Msc*I-*PALF2-R*	5'- TGG CCA TGC CAT CAT CTT GAC GAG -3'
MscI-PT7-rbs-4CL-F	5'- TGG CCA **TAATACG** **AAGG** TTC CGC AGC GAG TAC -3
*Bam*HI-4CL-R	5'- GGA TCC CTG AGC TGT CGG CGG AGG AT -3'
*Bam*HI-PT7-rbs-CHS-F	5'- GGA TCC **TAATACG** **AAGG** ACA TTT TTC TGG CAA AAA A -3'
*Sac*I-CHS-R	5'- GAG CTC AGT GCA CAA ACT GTG GAG C -3'
*Sac*I-PT7-rbs-CHI-F	5'- GAG CTC **TAATACG** **AAGG** ATG TCT CCC TCA CAG T -3'
*Hind*III-CHI-R	5'- AAG CTT TTC AGC AGC AGC AGC TGT C -3'
*Nde*I-PT7-rbs-F3H-F	5'- CAT ATG **TAATACG** **AAGG** ATG GCG CCA ACA ACA AC -3'
*Sac*I-F3H-R	5'- GAG CTC AGC AAA AAT CTC ATC AGT GC -3'
*Sac*I-PT7-rbs-DFR-F	5'- GAG CTC **TAATACG** **AAGG** ATC ATG AAA GAC TCT GTT GC -3'
*Hind*III-DFR-R	5'- AAG CTT AAC CTT GTT GCC ATT GAC A -3'
*Hind*III-PT7-rbs-LCR-F	5'- AAG CTT **TAATACG** **AAGG** ATG ACT GTG TTG GAA TCT G -3'
*Not*I-LCR-R	5'- GCG GCC GCA GCA CAC ATT GTG ATG G -3'

Yellow colour indicates restriction endonuclease enzyme sequences; green colour indicates PT7 sequences; yellow colour indicates rbs sequences
